# Reduced uterine perfusion pressure as a model for preeclampsia and fetal growth restriction in murine: a systematic review and meta-analysis

**DOI:** 10.1152/ajpheart.00056.2024

**Published:** 2024-05-17

**Authors:** Caren M. van Kammen, Seija E. L. Taal, Kimberley E. Wever, Joey P. Granger, A. Titia Lely, Fieke Terstappen

**Affiliations:** ^1^Division of Nanomedicine, Department CDL Research, University Medical Center Utrecht, Utrecht, The Netherlands; ^2^Department of Woman and Baby, University Medical Center Utrecht, Wilhelmina Children’s Hospital, Utrecht, The Netherlands; ^3^Department of Anesthesiology, Pain, and Palliative Medicine, Radboud University Medical Center, Nijmegen, The Netherlands; ^4^Department of Physiology and Biophysics, Cardiovascular-Renal Research Center, University of Mississippi Medical Center, Jackson, Mississippi, United States

**Keywords:** fetal growth restriction, placental insufficiency, preeclampsia, pregnancy, reduced uterine perfusion pressure

## Abstract

The reduced uterine perfusion pressure (RUPP) model is frequently used to study preeclampsia and fetal growth restriction. An improved understanding of influential factors might improve reproducibility and reduce animal use considering the variability in RUPP phenotype. We performed a systematic review and meta-analysis by searching Medline and Embase (until 28 March, 2023) for RUPP studies in murine. Primary outcomes included maternal blood pressure (BP) or proteinuria, fetal weight or crown-rump length, fetal reabsorptions, or antiangiogenic factors. We aimed to identify influential factors by meta-regression analysis. We included 155 studies. Our meta-analysis showed that the RUPP procedure results in significantly higher BP (MD = 24.1 mmHg; [22.6; 25.7]; *n* = 148), proteinuria (SMD = 2.3; [0.9; 3.8]; *n* = 28), fetal reabsorptions (MD = 50.4%; [45.5; 55.2]; *n* = 42), circulating soluble FMS-like tyrosine kinase-1 (sFlt-1) (SMD = 2.6; [1.7; 3.4]; *n* = 34), and lower fetal weight (MD = −0.4 g; [−0.47; −0.34]; *n* = 113. The heterogeneity (variability between studies) in primary outcomes appeared ≥90%. Our meta-regression identified influential factors in the method and time point of BP measurement, randomization in fetal weight, and type of control group in sFlt-1. The RUPP is a robust model considering the evident differences in maternal and fetal outcomes. The high heterogeneity reflects the observed variability in phenotype. Because of underreporting, we observed reporting bias and a high risk of bias. We recommend standardizing study design by optimal time point and method chosen for readout measures to limit the variability. This contributes to improved reproducibility and thereby eventually improves the translational value of the RUPP model.

## INTRODUCTION

Cardiovascular disease (CVD) is the primary cause of maternal deaths during pregnancy, with mounting evidence suggesting a significant link between pregnancy complications and the later development of CVD (1–3). Preeclampsia (PE) and fetal growth restriction (FGR) are such cardiovascular pregnancy complications that majorly impact maternal and fetal morbidity and mortality. Placental insufficiency forms the common etiology of these complex syndromes. Currently, there is no treatment available for this multifactorial disease. The search for a safe and effective therapy for this cardiovascular complication during pregnancy requires the use of animal models.

**Figure 1.**
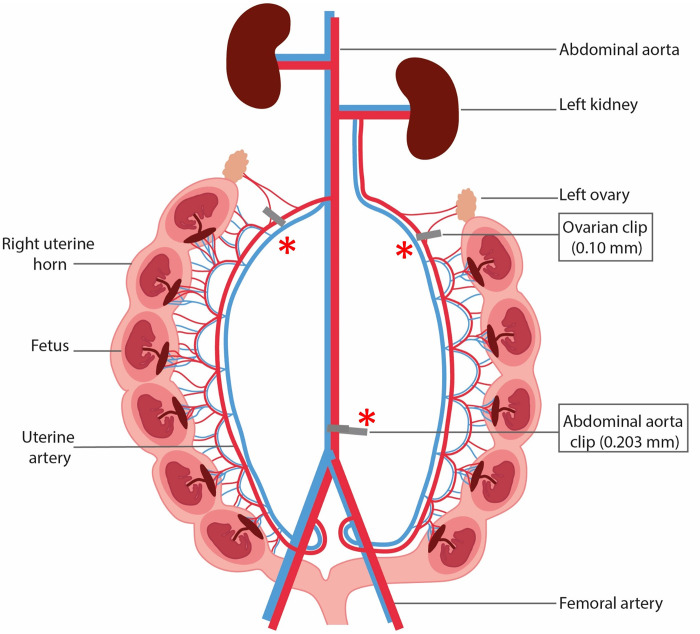
Schematic overview of reduced uterine perfusion pressure model. A surgical model induced in most cases (*) placing a clip (as illustrated in the picture) or a ligature around the abdominal aorta of a pregnant mouse or rat, below the renal arteries, during early to midgestation. The placement of a clip or ligature on the right and left ovarian arteries at the uterine arcade just before the first segmental artery prevents an adaptive increase in uterine blood flow via the ovarian artery.

A commonly used animal model to understand the pathophysiology of PE and FGR is the reduced uterine perfusion pressure (RUPP) (Fig. 1), a surgical model introduced by Granger et al. in 2006 (4), as a modification of the preexisting model by Eder and McDonald in 1987 (5). The induction occurs by placing a clip around a pregnant rat’s abdominal aorta, below the renal arteries, during early to midgestation. The placement of a clip on the right and left ovarian arteries at the uterine arcade just before the first segmental artery prevents an adaptive increase in uterine blood flow via the ovarian artery. Most studies applied the RUPP model in rats, but the procedure has also been adapted for several other species including mice, dogs, rabbits, sheep, and primates. The rat model mimics many of the prominent human characteristics of PE and FGR secondary to placental ischemia including proteinuria, endothelial dysfunction, hypertension in the mother, and restricted growth in the fetus (6). Furthermore, recent research shows the presence of cardiovascular and metabolic remodeling of the maternal and fetal heart (7–9). This suggests a strong association between preeclampsia and cardiovascular disease.

Murine (rats and mice) subjected to RUPP appears to have a variable degree of severity in the manifestation of PE/FGR phenotype. Multiple study characteristics potentially influence the response to RUPP. The extent to which these variables influence the severity of PE and FGR remains uncertain. The years of experience with the RUPP model in mainly rats have yielded an abundance of data that allow the identification of influential factors.

Accordingly, this systematic review and meta-analysis aimed to assess the impact of animal characteristics and methodological differences of the RUPP model on the preeclamptic and fetal growth-restricted phenotype. This may offer a perspective of influential factors on the heterogeneity (variability between studies) in phenotypical outcome and quality of published studies which could assist in optimizing the RUPP model and experimental design of future studies and thereby eventually improve the translational value of the RUPP model. Primary outcomes consist of maternal mean blood pressure or proteinuria, fetal weight or crown-rump length, and percentage of fetal reabsorptions; secondary outcome entails the level of the circulating soluble FMS-like tyrosine kinase-1 (sFlt-1) as the most studied antiangiogenic factor in PE. Furthermore, we set out to analyze influential factors by subgroup analysis, including species, strain, age, pairing details, method and time point of measurements, anesthesia during surgery and measurements, and the type of control group in the RUPP model.

## MATERIALS AND METHODS

### Study Protocol

This systematic review is reported according to the Preferred Reporting Items for Systematic Reviews and Meta-Analyses (PRISMA) (Supplemental Table S1). Before starting, we registered a review protocol for animal studies on PROSPERO (CRD42022335776). Key elements of this protocol on PROSPERO are described in the following paragraphs. The few amendments (Supplemental Table S2) to the preregistered protocol are reported in the Supplemental Data and referred to as post hoc analyses.

### Data Source and Search Strategy

On March 28, 2023, we performed a systematic literature search in the databases “Embase” and “Medline” through Ovid to identify studies using the RUPP model in murine which also reported on phenotypical outcomes of the preeclampsia and fetal growth restriction. No restrictions on language or publication date were applied (Supplemental Table S3). Search results were deduplicated using Rayyan (https://www.rayyan.ai/).

### Study Selection: Inclusion and Exclusion Criteria

Two independent researchers (Cv.K. and S.T.) screened the retrieved studies using Rayyan. Screening for eligibility was first performed based on title and abstract, followed by screening for final inclusion based on full text. In case of disagreement, a third independent investigator was consulted (K.W.). Studies were considered eligible when all of the following inclusion criteria applied: *1*) the study was a primary animal study; *2*) the study used the species of interest (rats or mice); *3*) the RUPP model was applied; *4*) a relevant control group was used; *5*) at least one of the outcome measures was reported: namely, maternal blood pressure (BP), proteinuria, percentage of fetal reabsorptions, fetal weight, fetal crown-to-rump length, and levels of circulating antiangiogenic factors. We defined the RUPP model as surgical induction by partially clamping both the abdominal aorta and the two ovarian arteries; other strategies of induction were excluded. We excluded publications that lacked original data (e.g., conference abstracts and reviews), as well as studies using genetically modified animals and those lacking an untreated control group and/or untreated RUPP group.

### Data Extraction

Two independent reviewers (Cv.K. and S.T.) extracted data in duplicate on subject characteristics (including species, strain, dam age, pairing, and time point of induction of RUPP), study design (including randomization, experimental unit fetus or dam, number of animals per group, number of animals per assay), method and timing of measurements. Our primary outcomes were maternal blood pressure (MAP, systolic BP, and diastolic BP), proteinuria, the percentage of fetal reabsorptions, fetal weight, and fetal crown-to-rump length. In addition, we extracted data on levels of circulating antiangiogenic factors as a secondary outcome. When outcome data were only presented in figures, we extracted them using a digital tool (http://automeris.io/WebPlotDigitizer/). For each outcome, we extracted means (SD) and number of subjects per experimental group.

### Assessment of Risk of Bias and Study Quality

Two reviewers (Cv.K. and S.T.) independently assessed the risk of bias for each included study, using SYRCLE’s risk of bias tool for animal studies (10). Both reviewers resolved discrepancies through discussion. If no consensus could be achieved, a third researcher (F.T.) served in two cases as an arbiter.

### Data Synthesis

Meta-analysis was performed when the prespecified required minimum of 20 studies was reached for an outcome, using the online R software (https://posit.co/cloud) with the meta and metafor package (v.4.2-0, Auckland, New Zealand). Meta-analysis methodology was performed according to gold-standard guidelines (11, 12). The mean difference (MD) and 95% confidence intervals (CIs) were calculated to compare the blood pressure in mmHg, fetal weight in grams, and fetal reabsorptions in percentage between control and RUPP groups. For blood pressure analysis, we used MAP and when not available SBP was used, a post hoc sensitivity analysis was performed to justify the pooling of the MAP and SBP.

The percentage of reabsorptions in some studies needed to be calculated from, e.g., the difference in litter size between *gestational day* (GD) *14* and GD19, or from the survival rate. In such cases, no SD could be recalculated because of the lack of individual data points. We therefore calculated the mean SD of all other comparisons and imputed this in the aforementioned cases. We assessed the robustness of this method by performing a post hoc sensitivity analysis in which all comparisons with an imputed SD were omitted from the analysis. Proteinuria and circulating antiangiogenic factor were synthesized using the standardized mean difference (SMD [95% CI]) to correct for the use of different units of measurements and assay kits.

When studies measured on multiple time points, we extracted all the measurements but eventually included only the most frequently reported time point for the outcome in the meta-analysis. In addition, a post hoc analysis of the type of control [sham vs. normal pregnant (NP)] was performed. For studies that used both NP and sham animals as control groups, only the sham group was included in the meta-analysis. In four rat studies, a correction of plug control in outcome measures was applied, i.e., the vaginal plug confirmation was reported as GD1 instead of GD0 or 0.5 and thereby extrapolating that the measurement also occurred a day earlier than described. In fetal weight analysis, we excluded studies using the individual fetus as the unit of analysis instead of the litter (*n* = 4).

To investigate potential sources of heterogeneity (stratified), meta-regression was performed on predefined subgrouping variables, provided that at least two subgroup categories contained at least 10 studies. The variables considered were (as per protocol) species (mice vs. rat); strain (Sprague–Dawley rat vs. Wistar rat vs. balb/c mouse vs. C57bl6 mouse vs. other); dam age [early young adults 8–10 wk vs. young adult 11–13 wk (i.e., at the plateau phase of their growth curve) vs. later age]; parity (primiparous vs. multiparous); time point of RUPP (early mouse GD <13 vs. standard 13 vs. late > 13); (early rat GD <14 vs. standard 14 vs. late > 14); method of blood pressure measurement [conscious restrained vs. conscious free (telemetry) vs. under anesthesia]; method of proteinuria measurement (spot vs. 24 h continuous measurement); time point of measurement at the gestational day of pregnancy (using in linear regression), and randomization (randomization vs. not reported vs. not randomized).

To account for expected between-study heterogeneity, a random effects model was used. A two-sided *P* value < 0.05 was considered significant for all overall analyses. For subgroup analysis, this significance level was adjusted for multiple comparisons using the Bonferroni–Holmes correction. To quantify the degree of heterogeneity across the included studies, the I^2^ statistic was used. The I^2^ statistic describes the degree of heterogeneity across studies in the outcome of the RUPP animal model, with the absence of heterogeneity being defined as 0%, whereas we declare >0–50, >50, and >80% as indicators of low, moderate, and high degrees of heterogeneity, respectively. Publication bias was investigated for each outcome reported in ≥20 studies, using visual inspection of funnel plots and Egger’s regression.

## RESULTS

### Study Selection and Overall Study Characteristics

**Figure 2.**
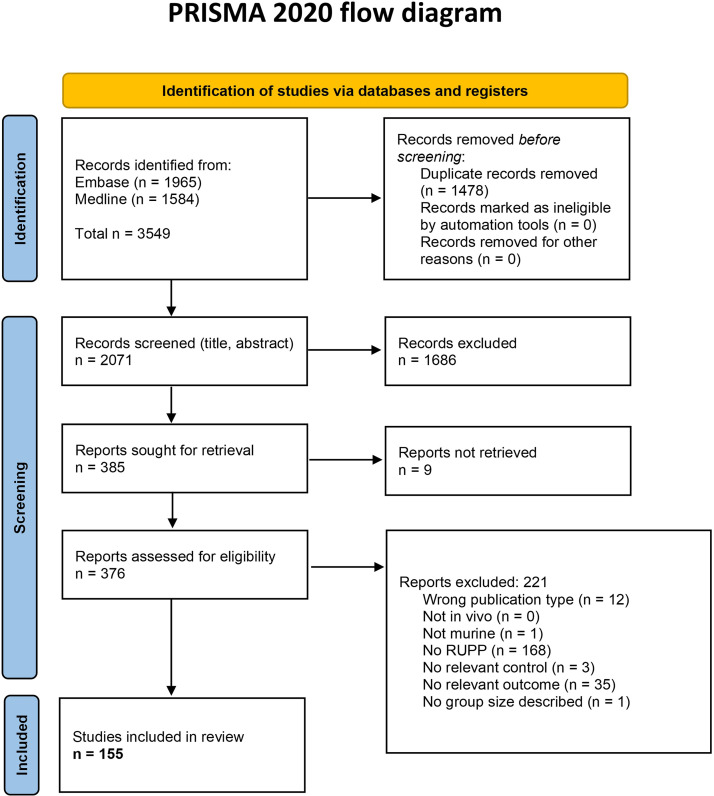
Flowchart of identification and selection process according to Preferred Reporting Items for systematic reviews and Meta-Analyses (PRISMA). The search strategy retrieved 2,071 unique hits via Embase and MEDLINE using OVID, of which we included 155 studies reporting relevant outcomes in the reduced uterine perfusion pressure (RUPP) model.

The search yielded a total of 2,071 studies after duplicate removal (Fig. 2). The majority of the exclusions (*n* = 376) after title and abstract screening were based on the use of a different animal model, or the record not being a primary animal study. Our analysis after full-text screening incorporated a total of 155 studies reporting relevant outcomes in the RUPP model. We performed meta-analyses on maternal blood pressure (BP), proteinuria, percentage of fetal reabsorptions, fetal weight (FW), and levels of circulating sFlt-1 as an antiangiogenic factor (Table 1). Data extraction per outcome is reported in a Supplemental Excel overview E1. The number of studies reporting fetal crown-to-rump length and other antiangiogenic factors did not meet the prespecified threshold of 20 studies required for meta-analysis.

**Table 1.** Overview of reported outcomes per included study using the RUPP model

Author (et al.), Year (Ref. No.)	Species, Strain	Type of Control	Maternal BP	Maternal Proteinuria	Fetal Reabsorptions	Fetal Weight	Circulating sFlt-1
Akhaphong, 2018 (13)	Rat, Sd	Sham	✓	–	✓	✓^1^	–
Alexander, 2001 (14)	Rat, Sd	Sham	✓	–		✓	–
Alexander, 2001 (15)	Rat, Sd	NP	✓	–	–	✓	–
Alexander, 2001 (16)	Rat, Sd	Sham	✓	✓	–	–	–
Alexander, 2004 (17)	Rat, Sd	Sham	✓	–	–	✓	–
Amaral, 2013 (18)	Rat, Wistar	Sham	✓	–	–	–	–
Amaral, 2015 (19)	Rat, Sd	NP	✓	–	–	✓	–
Amaral, 2017 (20)	Rat, Sd	NP	✓	–	–	✓	✓
Anderson, 2005 (21)	Rat, Sd	Sham	✓	–	–	✓	–
Ashraf, 2022 (22)	Rat, Sd	Sham	–	–	✓	✓	–
Bakrania, 2019 (23)	Rat, Sd	Sham	✓	–	–		✓
Balta, 2011 (24)	Rat, Sd	Sham	✓	✓	–	✓	–
Gilbert, 2012 (25)	Rat, Sd	NP	✓	–	–	✓	✓
Banek, 2013 (26)	Rat, Sd	NP and Sham^2^	✓	–	✓	✓	✓
Barron, 2001 (27)	Rat, Sd	Sham	✓	–	–	–	–
Bauer, 2013 (28)	Rat, Sd	Sham	✓	–	–	✓	✓
Brennan, 2016 (29)	Rat, Sd	Sham	✓	–	–	✓	–
Chang, 2005 (30)	Rat, Sd	Sham	✓	–	–	✓	–
Chatre, 2022 (31)	Rat, Sd	Sham	✓	–	–	✓^3^	–
Chen, 2008 (32)	Rat, Sd	Sham		–	–	✓	–
Chen, 2017 (33)	Rat, Sd	Sham	✓	–	–	✓	–
Chen, 2019 (34)	Rat, Sd	NP	✓	–	–	✓	–
Coats, 2021 (35)	Rat, Sd	Sham	–	–	–	✓^4^	–
Coats, 2021 (36)	Rat, Sd	Sham	–	–	✓	✓	–
Cornelius, 2013 (37)	Rat, Sd	NP	✓	–	–	✓	–
Cornelius, 2015 (38)	Rat, Sd	NP	✓	–	–	✓	–
Cornelius, 2016 (39)	Rat, Sd	NP	✓	–	–	✓	–
Cottrell, 2019 (40)	Rat, Sd	NP	✓	–	–	✓	–
Cottrell, 2021 (41)	Rat, Sd	NP	✓	–	–	✓	–
Crews, 2000 (42)	Rat, Sd	Sham	✓	–	–	–	–
Cunningham, 2018 (43)	Rat, Sd	NP	✓	–	–	✓	✓
Cunningham, 2020 (44)	Rat, Sd	NP	✓	–	–	✓	–
Cunningham, 2021 (45)	Rat, Sd	NP	✓	–	✓	✓	–
Darby, 2013 (46)	Rat, Sd	NP	✓	–	–	–	–
Deer, 2021 (47)	Rat, Sd	NP	✓	–	–	✓	–
Deer, 2021 (48)	Rat, Sd	NP	✓	–	✓	✓	–
Deng, 2022 (49)	Rat, Sd	Sham	✓	✓	✓	–	✓
Dias-Junior, 2017 (50)	Rat, Sd	Sham	✓	–	–	✓	✓
Ding, 2018 (51)	Rat, Sd	Sham	✓	–	–	–	–
Duncan, 2020 (52)	Rat, Sd	NP	✓	–	–	–	–
Eddy, 2020 (53)	Rat, Sd	Sham	✓	–	✓	✓	–
Elfarra, 2017 (54)	Rat, Sd	NP	✓	–	–	✓	–
Elfarra, 2020 (55)	Rat, Sd	NP and Sham^1^	✓	–	–	✓	–
El-Saka, 2019 (56)	Rat, Wistar	Sham	✓	✓	–	✓	✓
Cristóvão Escouto, 2018 (57)	Rat, Wistar	NP	✓	–	–	–	–
Faulkner, 2016 (58)	Rat, Sd	NP	✓	–	✓	✓	✓
Faulkner, 2018 (59)	Rat, Sd	NP	✓	–	–	–	–
Fraser, 2015 (60)	Rat, Sd	Sham	✓	–	–	–	–
Gadonski, 2006 (61)	Rat, Sd	NP	✓	–	–	–	–
George, 2011 (62)	Rat, Sd	NP	✓	–	–	✓	✓
George, 2013 (63)	Rat, Sd	NP	✓	–	–	✓	–
George, 2014 (64)	Rat, Sd	Sham	✓	–	–	✓	–
Giambrone, 2019 (65)	Rat, Sd	Sham	✓	–	–	✓	–
Giardina, 2002 (66)	Rat, Sd	Sham	✓	–	–	–	–
Gilbert, 2007 (67)	Rat, Sd	NP	✓	–	–	✓^3^	–
Gilbert, 2007 (68)	Rat, Sd	NP	✓	–	✓	✓	✓
Gilbert, 2009 (69)	Rat, Sd	NP	✓	–	–	✓	–
Gilbert, 2010 (70)	Rat, Sd	NP	✓	–	–	✓	✓
Gilbert, 2012 (71)	Rat, Sd	Sham	✓	–	–	✓	–
Gilbert, 2012 (25)	Rat, Sd	Sham	✓	–	–	✓	–
Gutkowska, 2011 (72)	Rat, Sd	NP	✓	–	–	–	–
Haase, 2020 (73)	Rat, Sd	Sham	✓	✓	–	✓	–
Han, 2018 (74)	Rat, Sd	Sham	✓	–	–	✓	✓
Harmon, 2015 (75)	Rat, Sd	NP	✓	–	–	✓	–
Hassanzadeh-Taheri, 2022 (76)	Rat, Wistar	Sham	✓	✓	–	✓^5^	–
Heltemes, 2010 (77)	Rat, Sd	NP	✓	–	–	✓	–
Herrock, 2023 (78)	Rat, Sd	NP	✓	–	✓	✓	–
Herrock, 2023 (79)	Rat, Sd	NP	✓	–	–	✓	–
Herse, 2012 (80)	Rat, Sd	NP	✓	–	–	✓	–
Hines, 2007 (81)	Rat, Sd	Sham	✓	–	✓	–	–
Huang, 2021 (82)	Rat, Sd	Sham	✓	–	–	✓	✓
Ibrahim, 2017 (83)	Rat, Sd	NP	✓	–	–	✓	–
Intapad, 2014 (84)	Mouse, C57BL/6J	Sham	✓	✓	✓	✓	✓
Isler, 2003 (85)	Rat, Sd	Sham	✓	–	–	✓	–
Issotina Zibrila, 2021 (86)	Rat, Sd	Sham	✓	✓	–	–	–
Issotina Zibrila, 2021 (87)	Rat, Sd	Sham	✓	✓	✓	✓	–
Javadian, 2013 (88)	Rat, Sd	Sham	✓	✓	–	✓	–
Joyner, 2007 (89)	Rat, Sd	Sham	✓	✓	–	✓^4^	–
Kiprono, 2013 (90)	Rat, Sd	NP	✓	–	✓	✓	–
LaMarca, 2005 (91)	Rat, Sd	NP	✓	–	–	–	–
LaMarca, 2008 (92)	Rat, Sd	NP	✓	–	–	–	–
LaMarca, 2008 (93)	Rat, Sd	NP	✓	–	–	✓	–
Lamarca, 2011 (94)	Rat, Sd	NP	✓	–	–	✓	–
Laule, 2017 (95)	Rat, Sd	Sham	✓	–	✓	✓	–
Laule, 2019 (96)	Rat, Sd	Sham	✓	–	–	✓	–
Lawrence, 2019 (97)	Rat, Sd	Sham	✓	✓	–	✓	–
Lawrence, 2022 (98)	Rat, Sd	NP	✓	✓	–	–	–
Li, 2017 (99)	Rat, Sd	Sham	✓	–	–	✓	–
Lillegard, 2013 (100)	Rat, Sd	Sham	✓	–	✓	–	✓
Lillegard, 2014 (101)	Rat, Sd	Sham	✓	–	✓	✓	–
Lin, 2020 (102)	Rat, Sd	Sham	✓	–	–	✓	–
Liu, 2022 (103)	Rat, Sd	NP and Sham^1^	✓	✓	–	✓	✓
Llinás, 2002 (104)	Rat, Sd	NP	✓	–	–	–	–
Alexander, 2004 (17)	Rat, Sd	Sham	✓	–	–	–	–
Logue, 2017 (105)	Rat, Sd	Sham	✓	✓	✓	✓	✓
Ma, 2017 (106)	Rat, Sd	Sham	✓	✓	–	–	✓
Porcello Marrone, 2014 (107)	Rat, Wistar	NP	✓	–	–	–	–
Mazzuca, 2014 (108)	Rat, Sd	Sham	✓	–	–	✓	–
Mazzuca, 2023 (109)	Rat, Sd	Sham	✓	–	–	–	–
McCarthy, 2011 (110)	Rat, Sd	NP	✓	✓	–	✓	✓
Moore, 2003 (111)	Rat, Sd	Sham	–	–	–	✓	–
Morton, 2012 (112)	Rat, Sd	Sham	✓	–	–	✓	–
Morton, 2015 (113)	Rat, Sd	Sham	✓	–	–	✓	–
Morton, 2019 (114)	Rat, Sd	Sham	✓	✓	✓	✓^4^	–
Murphy, 2015 (115)	Rat, Sd	Sham	✓	–	–	–	✓
Neves, 2008 (116)	Rat, Sd	Sham	✓	✓	✓	✓^4^	–
Novotny, 2012 (117)	Rat, Sd	NP	✓	–	–	✓	–
Novotny, 2013 (118)	Rat, Sd	NP	✓	–	–	✓	–
Ojeda, 2016 (119)	Rat, Sd	NP	–	–	–	✓	–
Ou, 2020 (120)	Rat, Wistar	NP and Sham^1^	✓	–	–	–	–
Paauw, 2017 (121)	Rat, Sd	NP	✓	✓	✓	✓	✓
Palei, 2021 (122)	Rat, Sd	NP	✓		✓	✓	–
Pang, 2023 (123)	Rat, unknown	NP	✓	–	–	–	–
Ramirez, 2011 (124)	Rat, Sd	NP	✓	–	✓	✓	–
Regal, 2016 (125)	Rat, Sd	NP	✓	–	–	✓	–
Laule, 2017 (95)	Rat, Sd	NP	✓	–	✓	✓	–
Regal, 2019 (126)	Rat, Sd	NP	✓	–	–	✓	–
Reho, 2011 (127)	Rat, Sd	NP	✓	–	✓	✓	–
Ren, 2018 (128)	Rat, Sd	NP	✓	–	–	✓	✓
Ren, 2021 (129)	Rat, Sd	NP	✓	–	–	✓	–
Richards, 2021 (8)	Rat, Sd	NP	✓	–	✓	✓	–
Ryan, 2011 (130)	Rat, Sd	NP	✓	–	–	–	–
Santiago-Font, 2016 (131)	Rat, Sd	NP	✓	–	–	✓	✓
Sedeek, 2008 (132)	Rat, Sd	Sham	✓	–	–	✓	–
Sholook, 2007 (133)	Rat, Sd	Sham	✓	–	–	–	–
Spradley, 2016 (134)	Rat, Sd	NP	✓	–	✓	✓	✓
Spradley, 2018 (135)	Rat, Wistar	Sham	✓	–	✓	✓	–
Spradley, 2019 (136)	Rat, Sd	Sham	✓	–	✓	✓	–
Sun, 2020 (137)	Rat, Sd	Sham	✓	–	✓	✓^3^	✓
Tam, 2011 (138)	Rat, Sd	NP	✓	–	✓	✓	–
Tian, 2016 (139)	Rat, Sd	Sham	✓	✓	✓	✓	–
Travis, 2020 (140)	Rat, Sd	NP	✓	–	✓	✓	✓
Travis, 2021 (141)	Rat, Sd	Sham	✓	–	–	✓ ^4^	✓
Travis, 2021 (142)	Rat, Sd	NP	✓	–	✓	✓	✓
Travis, 2021 (143)	Rat, Sd	Sham	✓	–	✓	✓	✓
Ushida, 2016 (144)	Rat, Sd	Sham	✓	✓	✓	✓^3^	✓
Vaka, 2018 (145)	Rat, Sd	NP	✓	–	–	✓	–
Vaka, 2019 (146)	Rat, Sd	NP	✓	–	–	–	–
Veillon, 2009 (147)	Rat, Sd	NP	✓	–	–	✓	–
Walsh, 2009 (148)	Rat, Sd	NP and Sham^1^	✓	–	–	✓	–
Walsh, 2012 (149)	Rat, Sd	NP	✓	–	–	✓	–
Wang, 2017 (33)	Rat, Sd	Sham	✓	✓	–	–	–
Wang, 2023 (150)	Rat, Sd	Sham	✓	–	✓	✓	–
Warrington, 2015 (151)	Rat, Sd	NP	✓	–	–	–	–
Li, 2014 (152)	Rat, Sd	Sham	✓	–	–	✓	–
Wei, 2021 (153)	Mouse, C57BL/6J	Sham	✓	–	–	–	–
Williamson, 2020 (154)	Rat, Sd	Sham	✓	✓	–	✓	✓
Yang, 2019 (155)	Rat, Sd	Sham	✓	✓	–	✓	–
Yang, 2023 (156)	Mouse, BALB/c	Sham	✓	✓	✓	–	✓
Younes, 2020 (157)	Rat, Sd	Sham	✓	–	✓	✓^4^	–
Zhang, 2016 (158)	Rat, Sd	NP	✓	–	✓	✓	–
Zhang, 2020 (159)	Rat, Sd	Sham	✓	✓	–	–	–
Zhang, 2021 (160)	Rat, Sd	Sham	✓	–	–	–	–
Zhang, 2022 (161)	Rat, Sd	N/S	–	–	–	✓	–
Zheng, 2022 (162)	Rat, Sd	Sham	✓	✓	✓	✓	–

Main study characteristics and reported outcomes of included studies in alphabetical order. BP, blood pressure; Sd, Sprague-Dawley; NP, normal pregnant; N/S, not specified; sFlt-1, soluble FMS-like tyrosine kinase-1; RUPP, reduced uterine perfusion pressure. ^1^Sex differences were pooled for meta-analysis. ^3^Reporting fetus as experimental unit data, not included in the meta-analysis. ^2^In the case of reporting both NP and sham as a control group in one study, we only used the sham data in meta-analysis. ^4^Authors also conducted measurements on fetal crown-to-rump length; however, because of the limited number of studies available, we were unable to perform a meta-analysis. ^5^Data were exclusively presented in Hassanzadeh-Taheri et al. (76), Fig. 2, as we did not have the capability to measure individual data points; consequently, these data were not included in the meta-analysis.

### Animal and Study Characteristics

In the 155 included studies, a comparison was made between the experimental group exposed to RUPP surgery versus the NP group (*n* = 70) or undergoing sham surgery (*n* = 80) (Supplemental Excel overview E1). Five studies used both NP and sham as a control group. Most studies used Sprague Dawley rats (Sd) (*n* = 145) and only a few used Wistar rats (*n* = 7), C57BL/6 mice (*n* = 2), or balb/c mice (*n* = 1). The median maternal age at the day of delivery for both rats and mice was 12 wk (range, 6– 16 wk; reported in *n* = 45). The median body weight in rats was 238 g (range, 180–312 g; reported in *n* = 34) and for mice, one study reported body weight (range, 18–22 g; reported in *n* = 1). In both species, the median age at day of surgery (reported in *n* = 6) lies at 12 wk (range, 9–22 wk). On the day of surgery, the median body weight reported was 225 g in rats (range, 220–362 g; reported in *n* = 18). Only a few studies (*n* = 16) described details on parity.

### Surgery Characteristics

The majority of RUPP surgeries were performed on GD14 (*n* = 137) in the rat studies (*n* = 152) and GD13 in two of the three mice studies (Supplemental Excel overview E1). In the four rat studies that reported vaginal plug conformation as GD1 instead of GD0, the RUPP surgery was corrected to GD13 instead of the reported GD14. Mainly, isoflurane (*n* = 126) was used as an anesthetic during the surgery, with other types of used anesthesia being chloral hydrate 10% (*n* = 2), ketamine and xylazine (*n* = 3), pentobarbital (*n* = 2), or unknown (*n* = 23). The RUPP model was mostly induced by placing a silver clip around the lower abdominal aorta and the ovarian arteries (*n* = 142). Still, others used surgical clips (no material described, *n* = 3), plastic clips (*n* = 1), silk sutures (*n* = 10), or did not report induction method (*n* = 2). The diameter of the restriction was reported in 146 studies with the used clip or suture inner diameter (ID) in mm. Predominantly, an ID of 0.203-mm abdominal aorta clip in rats was used and 0.100-mm clip for ovarian arteries, other IDs ranged from 0.023 to 0.33 mm for abdominal aorta and 0.06 mm to 0.33 for ovarian arteries. In two of the three mice studies, an abdominal aorta clip of ID 0.1 mm and ovarian arteries clips of 0.05 mm were used. After surgery, follow-up time ranged from 8 h to 5 days, with mainly the outcome end point on GD19 (range, GD17 and GD22) or delivery of pups for fetal growth restriction follow-up.

### Meta-Analysis and Meta-Regression per Outcome

Overall, our meta-analysis showed increased maternal blood pressure (BP), proteinuria, fetal reabsorptions, circulating sFlt-1, and a decrease in fetal weight per litter in RUPP compared with control (Table 2). We will discuss these results in more detail per outcome (see *Maternal blood pressure*, *Maternal proteinuria*, *Percentage of fetal reabsortions*, *Fetal growth restriction*, and *Circulating sFlt-1*).

**Table 2.** Overall effect of performed meta-analysis of included studies using the RUPP model

	No. Comp	Pooled Estimate (MD/SMD)	95% CI	I^2^, %	*P* Value	Egger’s Regression *P* Value
Maternal blood pressure, mmHg	148	24.1 MD	[22.6; 25.7]	92	<0.0001	0.11
Proteinuria	28	2.3 SMD	[0.9; 3.8]	92	<0.0021	0.54
Fetal reabsorptions (%)^1^	42	50.4 MD	[45.5; 55.2]	92	<0.0001	0.27
Fetal weight, g	113	−0.4 MD	[−0.47; −0.34]	90	<0.0001	0.0032
Circulating sFlt-1	34	2.6 SMD	[1.7; 3.4]	84	<0.0001	0.43

Values are pooled estimates expressed in mean difference (MD) or standardized mean difference (SMD) with a 95% confidence interval [CI] using a random effect model. RUPP, reduced uterine perfusion pressure. ^1^In nine studies, data with imputed SD of the mean SDs were used. No. Comp, experimental comparisons; I^2^, heterogeneity; sFlt-1, circulating soluble FMS-like tyrosine kinase-1.

#### Maternal blood pressure.

Our meta-analysis on blood pressure showed an increase of 24.1 mmHg [22.6; 25.7; I^2^ = 92%; *P* < 0.0001; Supplemental Fig. S1] in RUPP animals (*n* = 1,659) compared with control animals (*n* = 1,575). First and foremost, artery cannulation (*n* = 138) was applied to measure maternal BP in the 148 included studies.

**Figure 3.**
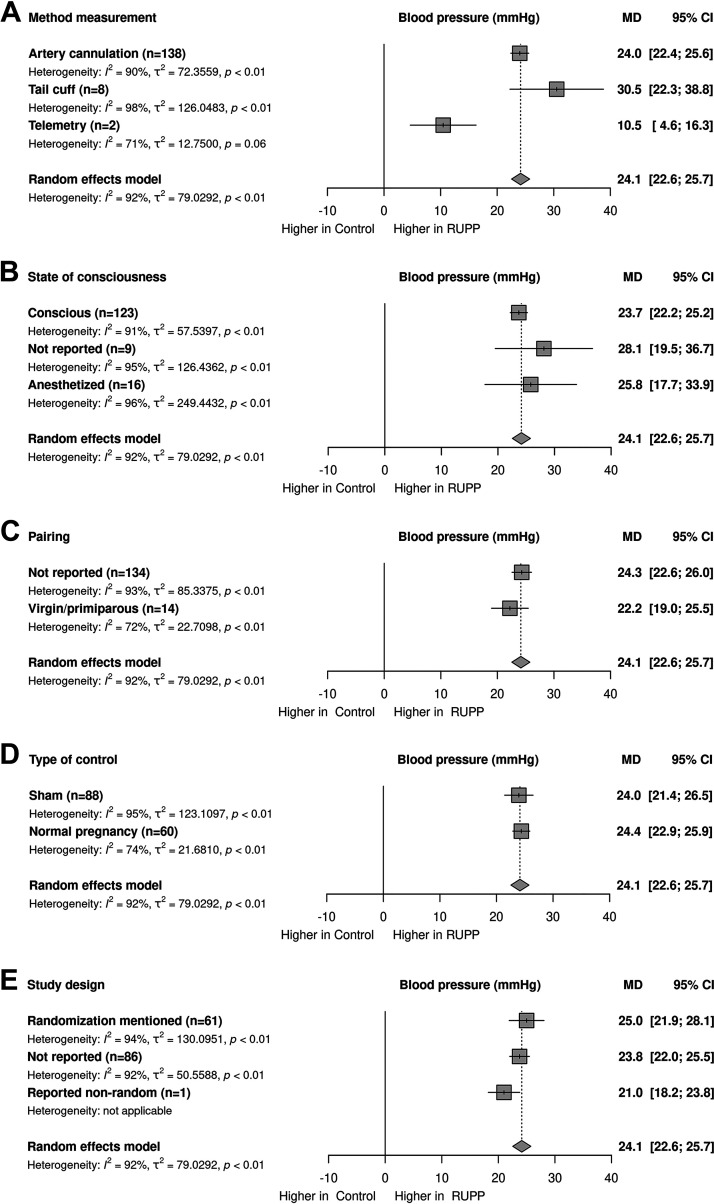
Stratified meta-regressions for the outcome of maternal blood pressure. *A*: method of blood pressure measurement. *B*: state of consciousness of animal during blood pressure measurement. *C*: pairing. *D*: type of control. *E*: study design on randomization. Data represent pooled estimates expressed as mean difference (MD) with a 95% confidence interval (CI) using a random effect model; *n*, number of independent comparisons in the stratum. RUPP, reduced uterine perfusion pressure.

**Figure 4.**
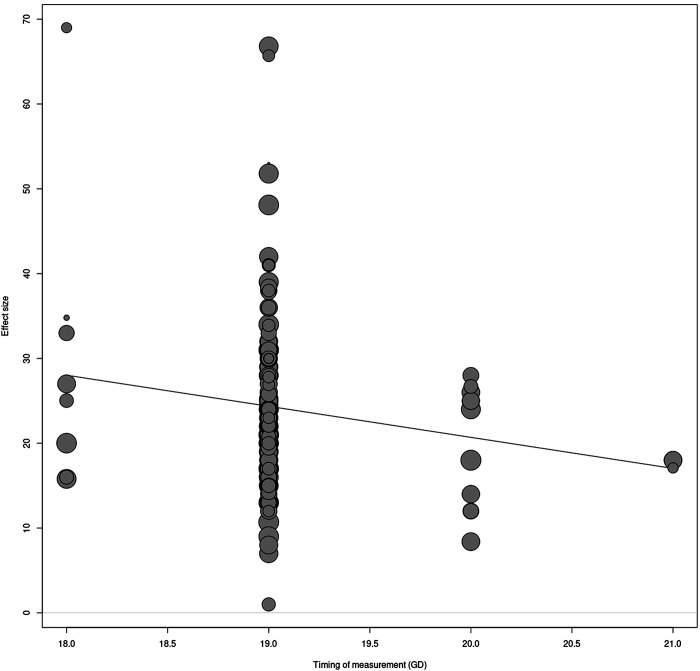
Time depending on the effect of maternal blood pressure measurement. Regression time-point bubble plot of maternal blood pressure measurement, time-point *gestational days* (GDs) *17–21*. On the time-points GD20 (*n* = 11) and GD21 (*n* = 3), effect of blood pressure (BP) is lower and revealed a significant regression *R*^2^ = 1.53%. *P* < 0.05.

We observed a significant effect in the meta-regression of the method of BP measurement [*R*^2^ = 5%; *P* < 0.01, Fig. 3*A*]; however, the effect is predominantly due to the much lower effect in the small stratum of two comparisons using telemetry. Predominantly, BP measurements were performed consciously (*n* = 120; of which CA is *n* = 110, tail-cuff *n* = 8, telemetry *n* = 2). In some of these cases, anesthesia was used on the day of BP measurement (*n* = 8) using isoflurane and in most cases after a minimal recovery of 1 h. Unconscious BP measurements under anesthesia (*n* = 16) were obtained in part of the studies that used artery cannulation. Meta-regression on the state of consciousness in animals during measurement showed no significant effect of this variable on the outcome (Fig. 3*B*). MAP is reported in most studies (*n* = 135). In other studies, SBP was used (*n* = 13), mainly the tail-cuff studies used SBP. Reporting the time point of BP at the gestational day of pregnancy was registered in 145 studies, the most used time point is GD19 (*n* = 123). A linear regression on time of measurement indicated a decrease in the effect of RUPP on BP over time [*R*^2^ = 1.53%; *P* < 0.05, Fig. 4]. Meta-regression on pairing (Fig. 3*C*); type of control (Fig. 3*D*); and reporting of randomization (Fig. 3*E*) showed no significant effect of these variables on the outcome.

Visual inspection of the funnel plots suggested no publication bias in BP (Supplemental Fig. S9*A*) which was supported by Egger’s regression test (Supplemental Fig. S9*B*).

#### Maternal proteinuria.

Meta-analysis on proteinuria showed increased levels of 2.3 of the SMD [0.9; 3.8; I^2^ = 92%; *P* = 0.0021; Supplemental Fig. S2] in animals subjected to RUPP (*n* = 301) referenced to control animals (*n* = 269), derived from 28 comparisons. Two different methods were used to collect urine samples, metabolic cage, or separate cage over 24-h collection (*n* = 17), or urine spot sample (*n* = 7) was collected directly from bladder, and four studies did not describe the collection method. To determine proteinuria, multiple assays were used, which measured concentrations of total protein (*n* = 14) or albumin (*n* = 3), or ratio of albumin and creatinine (*n* = 5), and five not specified assays.

The timing of proteinuria collection (varying from GD17 to GD20) did not influence the overall estimate (Supplemental Fig. S8*A*). Meta-regression of reporting of randomization (Supplemental Fig. S6) also showed no significant effect of this variable on the outcome.

Visual inspection of the funnel plots and Egger’s regression test suggested no publication bias in proteinuria (Supplemental Fig. S9, *C* and *D*).

#### Percentage of fetal reabsorptions.

The meta-analysis on the percentage of fetal reabsorptions showed an increase of 50.4% [45.5; 55.2; I^2^ = 92%; *P* < 0.0001; Supplemental Fig. S3] in dams subjected to RUPP (*n* = 520) compared with control dams (*n* = 458) based on 42 comparisons. Within this meta-analysis, most studies (*n* = 33) reported the exact percentages, as the difference between viable pups at GD14 and day of euthanization or as the differences in reabsorbed fetuses between GD14 and day of euthanization. In the cases where normal pregnant animals were used as the control group, researchers calculated implantation sites and reabsorption due to no surgery to determine the number of viable pups at GD14. In nine studies, the percentage of fetal reabsorptions had to be calculated because other ways of reporting were chosen.

Meta-regression on the timing of measurement (varying from GD18 to GD22) showed no effect of this variable on the outcome (Supplemental Fig. S8*B*). Meta-regression on type of control (Supplemental Fig. S7*A*) and reporting of randomization (Supplemental Fig. S7*B*) both showed no significant effects.

Visual inspection of the funnel plots suggested no publication bias in the percentage of fetal reabsorptions (Supplemental Fig. S9, *E* and *F*) which was confirmed by Egger’s regression test.

#### Fetal growth restriction.

The meta-analysis of fetal weight displayed a decrease of 0.4 g [−0.47; −0.34; I^2^ = 90%; *P* < 0.0001; Supplemental Fig. S4] in dams subjected to RUPP (*n* = 1,301) compared with control dams (*n* = 1,198) from a total of 113 comparisons. The decrease of 0.4-g MD is in line with our observation with a mean percent fetal weight loss of ∼16 ± 9% in RUPP compared with sham.

**Figure 5.**
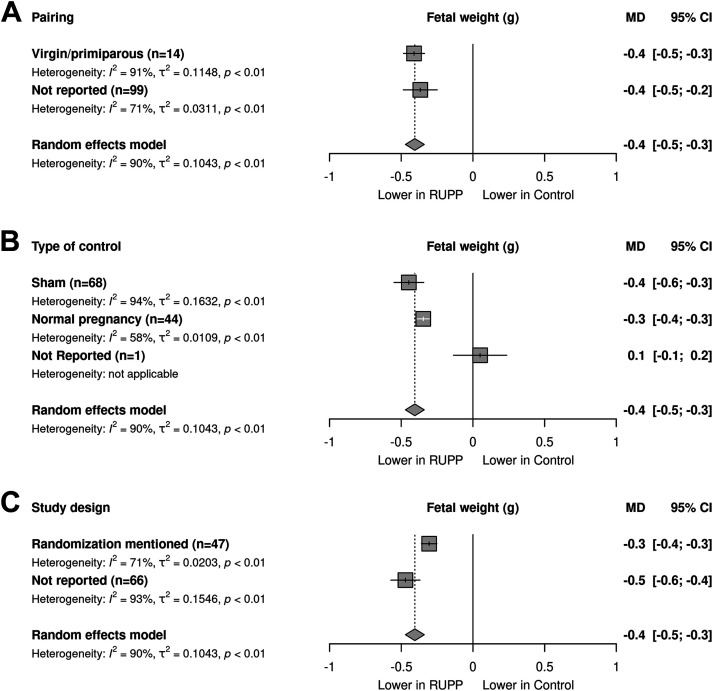
Stratified meta-regressions for the outcome of fetal weight. *A–C*: meta-regression on fetal weight as follows: pairing (*A*), type of control (*B*), and study design on randomization (*C*). Data represent pooled estimates expressed as mean difference (MD) with a 95% confidence interval (CI) using a random effect model; *n*, number of independent comparisons in the stratum. RUPP, reduced uterine perfusion pressure.

The time point of euthanasia to measure fetal weight showed a variation between GD18 and GD22, with the majority of the data reported on GD19 (*n* = 84); however, meta-regression did not reveal an effect of the timing on the outcome (Supplemental Fig. S8*C*). Meta-regression analysis revealed a smaller fetal weight difference between RUPP and control in studies that mentioned randomization versus those not reporting randomization [*R*^2^ = 3.9%; *P* < 0.024, Fig. 5*C*]. Meta-regressions on parity (Fig. 5*A*) and type of control (Fig. 5*B*) showed no effect of these variables on the outcome.

Visual inspection of the funnel plots suggested publication bias in fetal weight (Supplemental Fig. S9*K*); Egger’s regression test confirmed this (Supplemental Fig. S9*L*) (*P* < 0.0032, Table 2).

#### Circulating sFlt-1.

The meta-analysis of levels of sFlt-1, as the most studied antiangiogenic factor, showed an increased SMD of 2.6 [1.7; 3.4; I^2^ = 84%; *P* < 0.0001; Supplemental Fig. S5]. The meta-analysis combined results from the 155 independent studies from a total of 34 comparisons including animals subjected to RUPP (*n* = 343) compared with control animals (*n* = 332). All the reported studies used ELISA kits to measure sFlt-1 (*n* = 34), only the manufacturer of ELISA kits and the intra-assay and inter-assay precision with a coefficient of variability differed between studies or was not reported.

**Figure 6.**
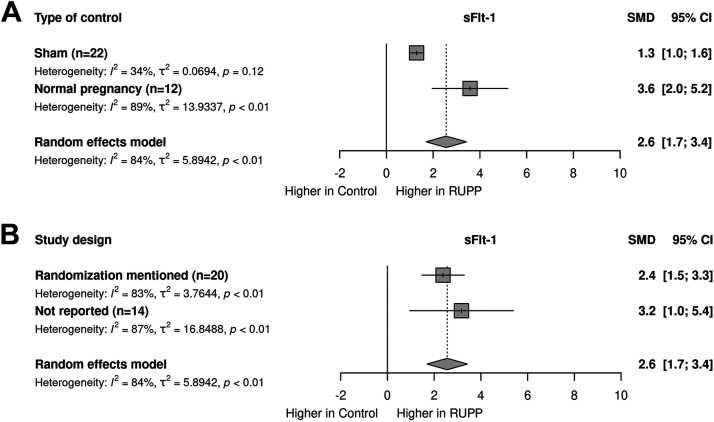
Stratified meta-regressions for the outcome circulating soluble FMS-like tyrosine kinase-1 (sFlt-1). *A*: type of control. *B*: study design on randomization. Data represent pooled estimates expressed as standardized mean difference (SMD) with a 95% confidence interval (CI) using a random effect model; *n*, number of independent comparisons in the stratum. RUPP, reduced uterine perfusion pressure.

Meta-regression on type of control demonstrated a higher effect in normal pregnant compared with sham animals [*R*^2^ = 5.2%; *P* < 0.05, Fig. 6*A*]. Meta-regression of the timing of measurement (Supplemental Fig. S8*D*) and study design reporting on randomization (Fig. 6*B*) showed no effect of these variables.

Visual inspection of the funnel plots suggested no publication bias in sFlt-1 (Supplemental Fig. S9*I*), which was supported by Egger’s regression test (Supplemental Fig. S9*J*).

#### Sensitivity analyses.

Two sensitivity analyses were performed to assess the influence of two important methodological decisions on the outcome of our meta-analyses. First, we assessed the effect of imputed SDs on the meta-analysis of fetal weight, by omitting all comparisons with an imputed SD from the analysis. Doing so did not significantly change the pooled estimate (original MD = 50.4% [45.5; 55.2]; I^2^ = 92% vs. sensitivity analysis MD = 49.7% [43.7; 55.6]; I^2^ = 93%). Subgroup analysis results remained unchanged (Supplemental Table S4).

Second, to assess the effect of our decision to pool MAP and SBP in the meta-analysis of blood pressure, we re-ran the analysis using MAP data only. Doing so did not significantly change the pooled estimate (original MD = 24.1 [22.6; 25.7]; I^2^ = 92% vs. sensitivity analysis SMD = 23.5 [22.0; 25.0]; I^2^ = 89%). Subgroup analysis results remained unchanged, except for the use of anesthetics during the blood pressure measurement, which was no longer significant. This is likely the result of collinearity between the blood pressure measurement method (tail-cuff vs. cannulation vs. telemetry), the use of anesthesia, and the type of measurement performed (MAP vs. SBP) (Supplemental Table S4).

### Reporting of Key Study Quality Indicators and Risks of Bias

**Figure 7.**
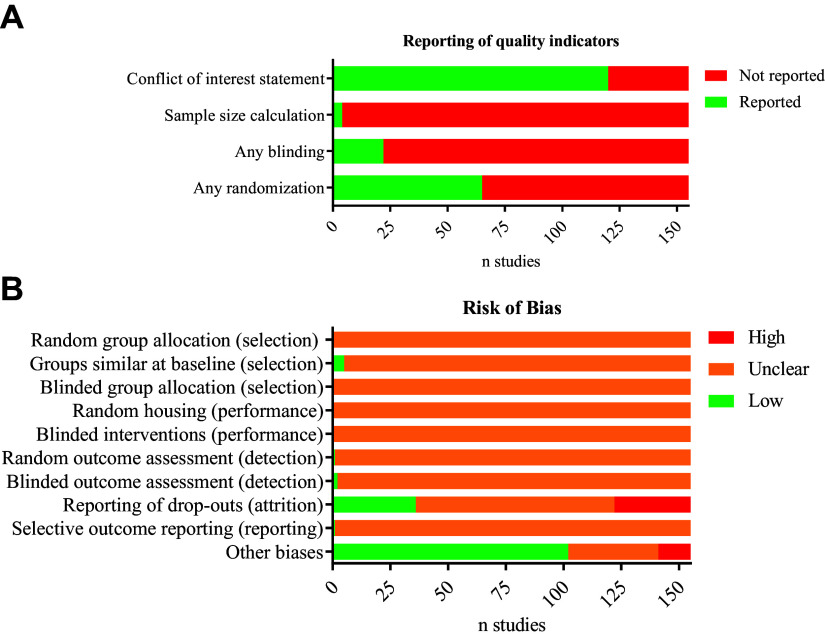
Risk of bias assessment. *A* and *B*: assessment of reporting of key study quality indicators (*A*) and risks of bias (*B*), according to SYRCLE’s risk of bias tool.

The reporting of key indicators of study quality showed ample room for improvement in most of the included articles (Fig. 7*A* and Supplemental Table S5). The majority of studies reported a conflict-of-interest statement (*n* = 120), where most of the authors declared to have no conflict of interest (*n* = 110). However, the vast majority failed to mention randomization (reported in *n* = 65), provide any information on a sample size calculation (reported in *n* = 4), or mention any blinding of the investigators during the study (reported in *n* = 22). Of note, reporting of blinding was even less prevalent for our predefined outcome measures (reported in *n* = 5).

Our assessment of internal validity shows that most studies are at unclear risk of several types of bias (Fig. 7*B* and Supplemental Table S5). This is mainly due to the absence of concrete measures to reduce bias and poor reporting of baseline characteristics of the animals. We considered all studies to be at unclear risk of performance bias for the domain “blinded interventions” (*item 5*) because no study mentioned blinding during this phase. However, this likely is an underestimation of the risk of bias in many cases (specifically for the non-sham control group), since the surgical procedures come with a visible postoperative appearance (sutures and effect on body wt) that sets RUPP apart from control animals. No study mentioned taking any extra precautions to prevent this bias (e.g., the surgeon not participating in subsequent phases of the experiment, or precautions to ensure blinding of the animal caretakers). A high risk of attrition bias (*item 8*) was observed in several studies because of unexplained dropouts. When considering other types of bias (*item 10*), we assessed studies at high risk of bias because of a possible conflict of interest (12 studies). We scored a high risk of bias in studies using the individual fetus as the unit of analysis instead of the litter (four studies). Using the individual pups as the unit of analysis is considered as a unit of analysis error since this artificially inflates the number of independent subjects in the analysis and can bias the results.

## DISCUSSION

This systematic review and meta-analysis support the use of the long-established murine RUPP model to study placental ischemia-induced PE and FGR. The maternal PE phenotype in RUPP is confirmed by significantly increased primary outcomes of hypertension and proteinuria, as well as the secondary outcome of circulating sFlt-1, together with the FGR phenotype through primary outcome of limited fetal growth and increased fetal resorptions. The presence of the high heterogeneity in all these outcomes makes it challenging to reproduce with a stable phenotype; however, this high heterogeneity reflects the complexities of the human condition. We discuss the observed heterogeneity in outcomes relating them to differences in characteristics and methods in *Influential Factors in Maternal Outcome* and *Influential Factors in Fetal Outcome*.

### Influential Factors in Maternal Outcome

#### Maternal blood pressure.

Our meta-analysis shows a significant increase of BP after RUPP induction, which is most pronounced in early pregnancy. The effect also appears to be modified by anesthetic use and the type and method of blood pressure measurement, but collinearity of these variables prevented us from pinpointing the contribution of each of these individual factors.

Although all BP measuring methods-, intra-arterial, telemetry, and tail-cuff, show a significant increase in BP in the RUPP model, the most optimal method is debatable and situation-dependent considering their specific advantages and disadvantages (163–165). The highest effect is measured in studies using tail-cuff, although also accompanied by the widest confidence interval. Tail-cuff is in general considered a much less reliable method to measure BP which requires adequate training of animals. Beyond that, our biggest concern in RUPP specifically is that the tail-cuff relies on sensor detection of blood flow instead of detection of the pulse (166, 167). Inherent to RUPP surgery, partly clamping of an artery affects distal flow through the tail, and thereby considerably impacts the tail-cuff BP measurements.

In the same line of reasoning, considering the clipping site, the carotid artery is the preferred and most reliable location for intra-arterial catheters in RUPP model to accurately measure blood pressure, surpassing the femoral artery. Altogether, it is logical that the majority of studies in our meta-analysis used carotid artery catheters. Although this method can detect small changes in BP, the drawback of this method lies in the need for anesthesia close to the time of recording (usually during or within 24 h of the measurement). Conjointly, analgesics are used to invasively insert the catheter, together with the administration of anticoagulants to keep the catheter patent. In our meta-regression, anesthesia during BP measurement was applied in only a small group of the intra-artery cannulation method (*n* = 16). It is known that each individual anesthetic has a unique impact on the cardiovascular system (168). The expected impact of anesthetics overall has not been observed in our meta-regression, possibly because of a diverse range of used anesthetics (*n* = 5), which cancel each other out. Moreover, we speculate that the anesthetic effect of each individual anesthetic might be divergent in control animals, which could be attributed to endothelial activation in the RUPP model. In addition, the effect of the model itself is among others reliant on the angiotensin II mechanism in the central nervous system (5).

The time point dependent effect on blood pressure matches the results of a within-study effect using telemetry (114). Both show a higher blood pressure difference during GD17 and GD19 compared with GD20 and GD21 in the rat RUPP model. This is most likely due to the first mechanistic effect of ischemic reperfusion diminishes by readaptation of vascular homeostasis (169). This also relates to healthy pregnancies, whereas the increased nitric oxide-mediated vasodilation results in decreased systemic vascular resistance and blood pressure in mid and late pregnancy (170–173).

Notably, the elevated effect in our meta-regression is much lower in telemetry compared with the other two methods. The use of telemetry allows us to accurately measure BP unrestrained and unanesthetized over a period of time, implying that factors such as anesthesia and stress of restrained animals influence measurement. Of note, the rat telemetry study showed only an increased blood pressure in RUPP during the active phase at night (114), highlighting the importance of timing of measurement during the day/night cycle. With this in mind and because of underreporting of the time of measurement, heterogeneity might be induced by variation of timing of measurement during the day, assuming measurements were conducted during working hours (inactive phase). Possibly, measuring BP in the active phase with a reversed day-night rhythm in a specific time window could improve reproducibility in future studies.

#### Maternal proteinuria.

Although the RUPP procedure results in proteinuria when looking at the overall meta-analysis, more than half of individual studies observed no effect and proteinuria also appears to be the phenotypical outcome with the highest variability. This is in line with the early observations described in the review by Granger et al. and comparable to the within-study variability of multiple time points (4, 24, 155, 156, 162). The variability might be caused by the variety of sampling methods and assays with different units of measurement. Another reason for the high between-study heterogeneity could be the relatively short exposure to placental ischemia used in the studies (maximum 5 days). Of note, the time window in the murine RUPP studies may be too short to measure an effect on proteinuria, as the RUPP procedure in baboons does not lead to an increase in protein excretion until *weeks 2* and *3* (174, 175). Unfortunately, the existing evidence is too heterogeneous to formulate advice on the most optimal assay or study design.

#### Circulating antiangiogenic factor sFlt-1.

We showed that the increase in sFlt-1 after RUPP is more pronounced in studies using normal pregnant animals as controls than in those using sham-operated animals. This difference might be linked to an inflammatory response to surgery. Rats that underwent sham surgery (including telemetry probe implantation) showed elevated levels of cytokines similar to the RUPP group (114), which might indicate that elevations of cytokines in both groups are likely due to a systemic inflammatory response to the surgeries. In turn, this could influence the expression of sFlt-1 in endothelial cells and placental trophoblasts (176–178). Moreover, inflammation activates neutrophils, which can release proteases and reactive oxygen species that may play a role in cleavage of Flt-1 and increase sFlt-1 levels (179–183). The very wide CI might be due to the higher resorption rate since placental ischemia induces a higher release of sFlt-1. Other possible causes of heterogeneity involve the many different manufacturers and the sensitivity of the ELISA kits. Standardization provides more insight into the absolute effect of sFlt-1 in the RUPP model.

### Influential Factors on Fetal Outcome

#### Fetal growth restriction.

The effect on weight loss in the RUPP model is a relatively large effect compared with the human condition (184). An overestimation of the effect might be in play considering the observed publication bias. In addition, selection bias may have led to unequal baseline characteristics of the animals in the RUPP and control groups, especially since we cannot rule out that animals were assigned to a group based on litter size on the day of surgery. Litter size can affect FW and was underreported as a baseline characteristic. The gestational day of the measured FW did not affect the outcome in our analysis; nevertheless, we speculate that later time points result in more accurate readings. This speculation is based on the understanding that fetuses tend to increase in weight as they develop over time. Therefore, measurements taken at later time points may provide more accurate readings of the absolute values. In addition, the longer exposure to the model at later time points could potentially result in a more pronounced difference in weight between the RUPP and control groups. This, combined with the already subtle differences, may allow for a more accurate measurement of the differences between the two groups.

Sex differences in the fetal outcome of the RUPP model could be relevant in fetal weight and eventually in long-term outcome effects. As reported, the placental response to oxidative stress appears to be different in males compared with females (185, 186). Only one study reported sex-specific fetal weight outcomes and showed no significant effect that males are more affected in the RUPP model, while an opposite trend in females is seen (13). The low reporting of sex differences could be due to difficulties in determining the sexes of small fetuses visually. This can be done easily by PCR as used in other studies (187).

#### Percentage of fetal resorptions.

Our meta-analysis showed a significant increase in the percentage of resorptions, with a high heterogeneity which could not be explained by any of our predefined subgrouping variables. The source of this heterogeneity therefore remains unclear, warranting further research. Theoretically, a varying amount of fetal reabsorptions could cause a higher level of inflammatory and antiangiogenic factors, which could in turn affect the severity of the RUPP model (188) Furthermore, a higher resorption rate could influence fetal growth in the RUPP model, assuming that the individual RUPP fetuses take in more of the available oxygen and nutrients when the litter size is smaller (189, 190).

### Modifiable Factors in Animal and Surgery Characteristics

Unfortunately, we could not evaluate all the possible modifiable factors (such as strain, parity, and clipping details) for every outcome measure, because of the small subgroups or poor reporting. These factors might explain (part of) the heterogeneity of the RUPP model.

### Strengths and Limitations

To the best of our knowledge, this is the first systematic review with meta-analysis in the RUPP model, which provides a complete overview of the maternal and fetal phenotypes. The relatively large sample size creates a high power in the meta-analysis which allows examination of many characteristics as possible influential factors. In BP, we were able to indicate two influential factors, namely, method and timing of BP measurement. One influential factor in sFlt-1 was indicated to the type of control and in FW randomization was indicated as an influential factor. Furthermore, our assessment of reporting of key study quality indicators and risks of bias reveals essential points of improvement for future research; namely, more rigorous implementation and reporting of vital experimental details and measures to reduce bias. Of note, the predominantly unclear risk of bias observed in the included studies may have led to an overestimation of effect sizes, which decreases the confidence we can have in the meta-analysis results. The subgroup analyses in particular must be regarded as hypothesis generating.

A limitation of this systematic review is that our assessment of sources of heterogeneity was hampered by the underreporting of certain study characteristics. This prevented us from performing all our predefined subgroup analyses for all outcomes. Although we investigated the RUPP effect only in the murine RUPP model, chosen specifically to reduce variables, investigation of the RUPP effect between species and between multiple RUPP methods would be worthwhile. However, both are not within the scope of this systematic review.

### Perspectives and Significance

The RUPP model is of relevant use in research on cardiovascular complications during pregnancy regarding PE and FGR, considering the evident differences in maternal and fetal outcomes between RUPP and control. The RUPP model provides insight into particular aspects of this complex and multifactorial cardiovascular disease and contributes to our understanding of PE. However, high heterogeneity is observed in the model, and several factors can influence the severity of the RUPP model. Knowing these influential factors and limitations could contribute to improving comprehension of findings in future research using the RUPP model. Of course, this depends on the research question as pathophysiological mechanistic questions require a broad phenotype conform to the human condition, whereas research questions on novel therapeutics would benefit from an animal model with high reproducibility and small heterogeneity to detect potential small effects without needing a large group of animals. Based on this systematic review and meta-analysis of the RUPP model, we formed recommendations about the influential factors and limitations that can be considered when designing a study with RUPP. First, we advise BP measurements performed with intra-artery cannulation in the carotid artery with readout time point under GD20 or telemetry method until at least GD20. Second, the use of NP animals as a control group is justifiable depending on the study design. Third, proteinuria in RUPP rat is not of additional value as an outcome measure for future research, as the proteinuria outcome resulted in not being the most stable outcome measure. Possibly only in large groups, it could give enough power to show increased proteinuria in RUPP. If you are specifically interested in proteinuria as an outcome, an alternative PE model could be chosen, such as the Dahl rat. Fourth, fetal weight should be considered in light of viable litter size and sexes of fetuses. Another consideration to explore is the genetic differences and sensitivity to the RUPP in Sd and Wistar animals. Lastly, we strongly recommend improving the reporting of study design, e.g., according to the ARRIVE (Animal Research Reporting of In Vivo Experiments) guidelines, to improve study quality and to reduce the risk of bias.

## SUPPLEMENTAL DATA

10.6084/m9.figshare.25452043 Supplemental Tables S1–S5, Supplemental Figs. S1–S9, and Supplemental Excel file E1: https://doi.org/10.6084/m9.figshare.25452043.

## GRANTS

This study was supported by Meer Kennis Minder Dieren Grant 40-42600-98-476, from Netherlands Organization for Health Research and Development (ZonMw).

## DISCLOSURES

No conflicts of interest, financial or otherwise, are declared by the authors.

## AUTHOR CONTRIBUTIONS

A.T.L. conceived and designed research; C.M.v.K. and S.E.L.T. performed experiments; C.M.v.K. and S.E.L.T. analyzed data; C.M.v.K., K.E.W., and F.T. interpreted results of experiments; C.M.v.K. and K.E.W. prepared figures; C.M.v.K. drafted manuscript; S.E.L.T., K.E.W., J.P.G., and F.T. edited and revised manuscript; S.E.L.T., K.E.W., J.P.G., A.T.L., and F.T. approved final version of manuscript.

## References

[1] Ramlakhan KP, Johnson MR, Roos-Hesselink JW. Pregnancy and cardiovascular disease. Nat Rev Cardiol 17: 718–731, 2020. doi:10.1038/S41569-020-0390-Z. 32518358

[2] Duley L. The global impact of pre-eclampsia and eclampsia. Semin Perinatol 33: 130–137, 2009. doi:10.1053/J.SEMPERI.2009.02.010. 19464502

[3] Bellamy L, Casas JP, Hingorani AD, Williams DJ. Pre-eclampsia and risk of cardiovascular disease and cancer in later life: systematic review and meta-analysis. BMJ 335: 974–977, 2007. doi:10.1136/BMJ.39335.385301.BE. 17975258 PMC2072042

[4] Granger JP, LaMarca BBD, Cockrell K, Sedeek M, Balzi C, Chandler D, Bennett W. Reduced uterine perfusion pressure (RUPP) model for studying cardiovascular-renal dysfunction in response to placental ischemia. In: Placenta and Trophoblast New Jersey: Humana Press, 2006, p. 381–392. 10.1385/1-59259-989-3:381 16511995

[5] Eder DJ, McDonald MT. A role for brain angiotensin II in experimental pregnancy-induced hypertension in laboratory rats. Clin Exp Hypertens B 6: 431–451, 1987. doi:10.3109/10641958709023492.

[6] Li J, Lamarca B, Reckelhoff JF. A model of preeclampsia in rats: the reduced uterine perfusion pressure (RUPP) model. Am J Physiol Heart Circ Physiol 303: H1–H8, 2012. doi:10.1152/ajpheart.00117.2012. 22523250 PMC3404644

[7] Booz GW, Kennedy D, Bowling M, Robinson T, Azubuike D, Fisher B, Brooks K, Chinthakuntla P, Hoang NH, Hosler JP, Cunningham MW. Angiotensin II type 1 receptor agonistic autoantibody blockade improves postpartum hypertension and cardiac mitochondrial function in rat model of preeclampsia. Biol Sex Differ 12: 58, 2021. doi:10.1186/S13293-021-00396-X. 34727994 PMC8562001

[8] Richards C, Sesperez K, Chhor M, Ghorbanpour S, Rennie C, Ming CLC, Evenhuis C, Nikolic V, Orlic NK, Mikovic Z, Stefanovic M, Cakic Z, McGrath K, Gentile C, Bubb K, McClements L. Characterisation of cardiac health in the reduced uterine perfusion pressure model and a 3D cardiac spheroid model, of preeclampsia. Biol Sex Differ 12: 31, 2021. doi:10.1186/S13293-021-00376-1. 33879252 PMC8056582

[9] McClements L, Richards C, Patel N, Chen H, Sesperez K, Bubb KJ, Karlstaedt A, Aksentijevic D. Impact of reduced uterine perfusion pressure model of preeclampsia on metabolism of placenta, maternal and fetal hearts. Sci Rep 12: 1111, 2022. doi:10.1038/S41598-022-05120-2. 35064159 PMC8782944

[10] Hooijmans CR, Rovers MM, Bm De Vries R, Leenaars M, Ritskes-Hoitinga M, Langendam MW. SYRCLE’s risk of bias tool for animal studies. BMC Med Res Methodol 14: 43, 2014. doi:10.1186/1471-2288-14-43. 24667063 PMC4230647

[11] Vesterinen HM, Sena ES, Egan KJ, Hirst TC, Churolov L, Currie GL, Antonic A, Howells DW, Macleod MR. Meta-analysis of data from animal studies: a practical guide. J Neurosci Methods 221: 92–102, 2014 [Erratum in J Neurosci Methods 259: 156, 2016]. doi:10.1016/J.JNEUMETH.2013.09.010. 24099992

[12] Hooijmans CR, IntHout J, Ritskes-Hoitinga M, Rovers MM. Meta-analyses of animal studies: an introduction of a valuable instrument to further improve healthcare. ILAR J 55: 418–426, 2014. doi:10.1093/ILAR/ILU042. 25541544 PMC4276598

[13] Akhaphong B, Lockridge A, Jo S, Mohan R, Wilcox JA, Wing CR, Regal JF, Alejandro EU. Reduced uterine perfusion pressure causes loss of pancreatic β-cell area but normal function in fetal rat offspring. Am J Physiol Regul Integr Comp Physiol 315: R1220–R1231, 2018. doi:10.1152/AJPREGU.00458.2017. 30303709 PMC6425640

[14] Alexander BT, Cockrell K, Cline FD, Llinas MT, Sedeek M, Granger JP. Effect of angiotensin II synthesis blockade on the hypertensive response to chronic reductions in uterine perfusion pressure in pregnant rats. Hypertension 38: 742–745, 2001. doi:10.1161/01.HYP.38.3.742. 11566968

[15] Alexander BT, Rinewalt AN, Cockrell KL, Massey MB, Bennett WA, Granger JP. Endothelin type a receptor blockade attenuates the hypertension in response to chronic reductions in uterine perfusion pressure. Hypertension 37: 485–489, 2001. doi:10.1161/01.HYP.37.2.485. 11230323

[16] Alexander BT, Kassab SE, Miller MT, Abram SR, Reckelhoff JF, Bennett WA, Granger JP. Reduced uterine perfusion pressure during pregnancy in the rat is associated with increases in arterial pressure and changes in renal nitric oxide. Hypertension 37: 1191–1195, 2001. doi:10.1161/01.HYP.37.4.1191. 11304523

[17] Alexander BT, Llinas MT, Kruckeberg WC, Granger JP. L-arginine attenuates hypertension in pregnant rats with reduced uterine perfusion pressure. Hypertension 43: 832–836, 2004. doi:10.1161/01.HYP.0000119192.32360.a9. 14769812

[18] Amaral LM, Pinheiro LC, Guimaraes DA, Palei ACT, Sertório JT, Portella RL, Tanus-Santos JE. Antihypertensive effects of inducible nitric oxide synthase inhibition in experimental pre-eclampsia. J Cell Mol Med 17: 1300–1307, 2013. doi:10.1111/JCMM.12106. 23890248 PMC4159028

[19] Amaral LM, Cornelius DC, Harmon A, Moseley J, Martin JN, LaMarca B. 17-hydroxyprogesterone caproate significantly improves clinical characteristics of preeclampsia in the reduced uterine perfusion pressure rat model. Hypertension 65: 225–231, 2015. doi:10.1161/HYPERTENSIONAHA.114.04484. 25368030 PMC4350787

[20] Amaral LM, Faulkner JL, Elfarra J, Cornelius DC, Cunningham MW, Ibrahim T, Vaka VR, McKenzie J, LaMarca B. Continued investigation into 17-OHPC: results from the preclinical RUPP rat model of preeclampsia. Hypertension 70: 1250–1255, 2017. doi:10.1161/HYPERTENSIONAHA.117.09969. 29084881 PMC5901972

[21] Anderson CM, Lopez F, Zhang HY, Pavlish K, Benoit JN. Reduced uteroplacental perfusion alters uterine arcuate artery function in the pregnant Sprague-Dawley rat. Biol Reprod 72: 762–766, 2005. doi:10.1095/BIOLREPROD.104.036715. 15564595

[22] Ashraf UM, Hall DL, Campbell N, Waller JP, Rawls AZ, Solise D, Cockrell K, Bidwell GL, Romero DG, Ojeda NB, LaMarca B, Alexander BT. Inhibition of the AT1R agonistic autoantibody in a rat model of preeclampsia improves fetal growth in late gestation. Am J Physiol Regul Integr Comp Physiol 323: R670–R681, 2022. doi:10.1152/AJPREGU.00122.2022. 36121142 PMC9602704

[23] Bakrania BA, Hall ME, Shahul S, Granger JP. The reduced uterine perfusion pressure (RUPP) rat model of preeclampsia exhibits impaired systolic function and global longitudinal strain during pregnancy. Pregnancy Hypertens 18: 169–172, 2019. doi:10.1016/J.PREGHY.2019.10.001. 31669926 PMC7063592

[24] Balta O, Boztosun A, Deveci K, Gulturk S, Ekici F, Kaya A, Cetin A, Cetin M. Reduced uterine perfusion pressure model is not successful to mimic severe preeclampsia. Placenta 32: 675–680, 2011. doi:10.1016/J.PLACENTA.2011.06.005. 21722954

[25] Gilbert JS, Banek CT, Bauer AJ, Gingery A, Needham K. Exercise training attenuates placental ischemia-induced hypertension and angiogenic imbalance in the rat. Hypertension 60: 1545–1551, 2012. doi:10.1161/HYPERTENSIONAHA.112.202275. 23090773 PMC3909775

[26] Banek CT, Bauer AJ, Needham KM, Dreyer HC, Gilbert JS. AICAR administration ameliorates hypertension and angiogenic imbalance in a model of preeclampsia in the rat. Am J Physiol Heart Circ Physiol 304: H1159–H1165, 2013. doi:10.1152/AJPHEART.00903.2012. 23417865 PMC3625906

[27] Barron LA, Giardina JB, Granger JP, Khalil RA. High-salt diet enhances vascular reactivity in pregnant rats with normal and reduced uterine perfusion pressure. Hypertension 38: 730–735, 2001. doi:10.1161/01.HYP.38.3.730. 11566966

[28] Bauer AJ, Banek CT, Needham K, Gillham H, Capoccia S, Regal JF, Gilbert JS. Pravastatin attenuates hypertension, oxidative stress, and angiogenic imbalance in rat model of placental ischemia-induced hypertension. Hypertension 61: 1103–1110, 2013. doi:10.1161/HYPERTENSIONAHA.111.00226. 23460290 PMC3909776

[29] Brennan L, Morton JS, Quon A, Davidge ST. Postpartum vascular dysfunction in the reduced uteroplacental perfusion model of preeclampsia. PLoS One 11: e0162487, 2016. doi:10.1371/JOURNAL.PONE.0162487. 27658290 PMC5033327

[30] Chang EY, Barbosa E, Paintlia MK, Singh A, Singh I. The use of N-acetylcysteine for the prevention of hypertension in the reduced uterine perfusion pressure model for preeclampsia in Sprague-Dawley rats. Am J Obstet Gynecol 193: 952–956, 2005. doi:10.1016/J.AJOG.2005.05.083. 16157093

[31] Chatre L, Ducat A, Spradley FT, Palei AC, Chéreau C, Couderc B, Thomas KC, Wilson AR, Amaral LM, Gaillard I, Méhats C, Lagoutte I, Jacques S, Miralles F, Batteux F, Granger JP, Ricchetti M, Vaiman D. Increased NOS coupling by the metabolite tetrahydrobiopterin (BH4) reduces preeclampsia/IUGR consequences. Redox Biol 55: 102406, 2022. doi:10.1016/J.REDOX.2022.102406. 35964341 PMC9389306

[32] Chen W, Khalil RA. Differential [Ca2+]i signaling of vasoconstriction in mesenteric microvessels of normal and reduced uterine perfusion pregnant rats. Am J Physiol Regul Integr Comp Physiol 295: R1962–R1972, 2008. doi:10.1152/AJPREGU.90523.2008. 18843089 PMC2685295

[33] Wang C, Liu X, Kong D, Qin X, Li Y, Teng X, Huang X. Apelin as a novel drug for treating preeclampsia. Exp Ther Med 14: 5917–5923, 2017. doi:10.3892/ETM.2017.5304. 29250138 PMC5729370

[34] Chen H, Li J, Li N, Liu H, Tang J. Increased circulating trimethylamine N-oxide plays a contributory role in the development of endothelial dysfunction and hypertension in the RUPP rat model of preeclampsia. Hypertens Pregnancy 38: 96–104, 2019. doi:10.1080/10641955.2019.1584630. 30821524

[35] Coats LE, Bakrania BA, Bamrick-Fernandez DR, Ariatti AM, Rawls AZ, Ojeda NB, Alexander BT. Soluble guanylate cyclase stimulation in late gestation does not mitigate asymmetric intrauterine growth restriction or cardiovascular risk induced by placental ischemia in the rat. Am J Physiol Heart Circ Physiol 320: H1923–H1934, 2021. doi:10.1152/AJPHEART.00033.2021. 33739156 PMC8428889

[36] Coats LE, Bamrick-Fernandez DR, Ariatti AM, Bakrania BA, Rawls AZ, Ojeda NB, Alexander BT. Stimulation of soluble guanylate cyclase diminishes intrauterine growth restriction in a rat model of placental ischemia. Am J Physiol Regul Integr Comp Physiol 320: R149–R161, 2021. doi:10.1152/AJPREGU.00234.2020. 33175587 PMC7948129

[37] Cornelius DC, Hogg JP, Scott J, Wallace K, Herse F, Moseley J, Wallukat G, Dechend R, La Marca B. Administration of interleukin-17 soluble receptor C suppresses TH17 cells, oxidative stress, and hypertension in response to placental ischemia during pregnancy. Hypertension 62: 1068–1073, 2013. doi:10.1161/HYPERTENSIONAHA.113.01514. 24060899 PMC3899693

[38] Cornelius DC, Amaral LM, Harmon A, Wallace K, Thomas AJ, Campbell N, Scott J, Herse F, Haase N, Moseley J, Wallukat G, Dechend R, LaMarca B. An increased population of regulatory T cells improves the pathophysiology of placental ischemia in a rat model of preeclampsia. Am J Physiol Regul Integr Comp Physiol 309: R884–R891, 2015. doi:10.1152/AJPREGU.00154.2015. 26290102 PMC4666948

[39] Cornelius DC, Amaral LM, Wallace K, Campbell N, Thomas AJ, Scott J, Herse F, Wallukat G, Dechend R, LaMarca B. Reduced uterine perfusion pressure T-helper 17 cells cause pathophysiology associated with preeclampsia during pregnancy. Am J Physiol Regul Integr Comp Physiol 311: R1192–R1199, 2016. doi:10.1152/ajpregu.00117.2016. 27784685 PMC5256975

[40] Cottrell JN, Amaral LM, Harmon A, Cornelius DC, Cunningham MW, Vaka VR, Ibrahim T, Herse F, Wallukat G, Dechend R, Lamarca B. Interleukin-4 supplementation improves the pathophysiology of hypertension in response to placental ischemia in RUPP rats. Am J Physiol Regul Integr Comp Physiol 316: R165–R171, 2019. doi:10.1152/AJPREGU.00167.2018. 30624978 PMC6397356

[41] Cottrell JN, Witcher AC, Comley K, Cunningham MW, Ibrahim T, Cornelius DC, LaMarca B, Amaral LM. Progesterone-induced blocking factor improves blood pressure, inflammation, and pup weight in response to reduced uterine perfusion pressure (RUPP). Am J Physiol Regul Integr Comp Physiol 320: R719–R727, 2021. doi:10.1152/AJPREGU.00152.2020. 33533305 PMC8163613

[42] Crews JK, Herrington JN, Granger JP, Khalil RA. Decreased endothelium-dependent vascular relaxation during reduction of uterine perfusion pressure in pregnant rat. Hypertension 35: 367–372, 2000. doi:10.1161/01.HYP.35.1.367. 10642326

[43] Cunningham MW, Castillo J, Ibrahim T, Cornelius DC, Campbell N, Amaral L, Vaka VR, Usry N, Williams JM, LaMarca B. AT1-AA (angiotensin II type 1 receptor agonistic autoantibody) blockade prevents preeclamptic symptoms in placental ischemic rats. Hypertension 71: 886–893, 2018. doi:10.1161/HYPERTENSIONAHA.117.10681. 29555668 PMC5903585

[44] Cunningham MW, Jayaram A, Deer E, Amaral LM, Vaka VR, Ibrahim T, Cornelius DC, LaMarca B. Tumor necrosis factor alpha (TNF-α) blockade improves natural killer cell (NK) activation, hypertension, and mitochondrial oxidative stress in a preclinical rat model of preeclampsia. Hypertens Pregnancy 39: 399–404, 2020. doi:10.1080/10641955.2020.1793999. 32646252 PMC7669709

[45] Cunningham MW, Amaral LM, Campbell NE, Cornelius DC, Ibrahim T, Vaka VR, LaMarca B. Investigation of interleukin-2-mediated changes in blood pressure, fetal growth restriction, and innate immune activation in normal pregnant rats and in a preclinical rat model of preeclampsia. Biol Sex Differ 12: 4, 2021. doi:10.1186/S13293-020-00345-0. 33407826 PMC7789596

[46] Darby MM, Wallace K, Cornelius D, Chatman KT, Mosely JN, Martin JN, Purser CA, Baker RC, Owens MT, Lamarca BB. Vitamin D supplementation suppresses hypoxia-stimulated placental cytokine secretion, hypertension and CD4+ T cell stimulation in response to placental ischemia. Med J Obstet Gynecol 1: 1012, 2013. 25414911 PMC4235666

[47] Deer E, Reeve KE, Amaral L, Vaka VR, Franks M, Campbell N, Fitzgerald S, Herrock O, Ibrahim T, Cornelius D, LaMarca B. CD4+ T cells cause renal and placental mitochondrial oxidative stress as mechanisms of hypertension in response to placental ischemia. Am J Physiol Renal Physiol 320: F47–F54, 2021. doi:10.1152/AJPRENAL.00398.2020. 33196321 PMC7847053

[48] Deer E, Amaral LM, Campbell N, Fitzgerald S, Herrock O, Ibrahim T, Lamarca B. Low dose of IL-2 normalizes hypertension and mitochondrial function in the RUPP rat model of placental ischemia. Cells 10: 2797, 2021. doi:10.3390/CELLS10102797. 34685775 PMC8534834

[49] Deng Y, Lai W, Yu L, Zhang W, Ding Y. miR-2115-3p inhibits ferroptosis by downregulating the expression of glutamic-oxaloacetic transaminase in preeclampsia. Placenta 129: 94–103, 2022. doi:10.1016/J.PLACENTA.2022.09.014. 36279730

[50] Dias-Junior CA, Chen J, Cui N, Chiang CL, Zhu M, Ren Z, Possomato-Vieira JS, Khalil RA. Angiogenic imbalance and diminished matrix metalloproteinase-2 and -9 underlie regional decreases in uteroplacental vascularization and feto-placental growth in hypertensive pregnancy. Biochem Pharmacol 146: 101–116, 2017. doi:10.1016/J.BCP.2017.09.005. 28912068 PMC5705464

[51] Ding L, Bai C, Liu Y. Interleukin-6 contributes to myocardial damage in pregnant rats with reduced uterine perfusion pressure. Braz J Med Biol Res 51: e6921, 2018. doi:10.1590/1414-431X20186921. 29898033 PMC6002145

[52] Duncan JW, Azubuike D, Booz GW, Fisher B, Williams JM, Fan F, Ibrahim T, LaMarca B, Cunningham MW. Angiotensin II type 1 receptor autoantibody blockade improves cerebral blood flow autoregulation and hypertension in a preclinical model of preeclampsia. Hypertens Pregnancy 39: 451–460, 2020. doi:10.1080/10641955.2020.1833215. 33119997

[53] Eddy AC, Howell JA, Chapman H, Taylor E, Mahdi F, George EM, Bidwell GL. Biopolymer-delivered, maternally sequestered NF-κB (nuclear factor-κB) inhibitory peptide for treatment of preeclampsia. Hypertension 75: 193–201, 2020. doi:10.1161/HYPERTENSIONAHA.119.13368. 31786977 PMC7008946

[54] Elfarra J, Amaral LM, McCalmon M, Scott JD, Cunningham MW, Gnam A, Ibrahim T, LaMarca B, Cornelius DC. Natural killer cells mediate pathophysiology in response to reduced uterine perfusion pressure. Clin Sci (Lond) 131: 2753–2762, 2017. doi:10.1042/CS20171118. 29042488 PMC5864106

[55] Elfarra JT, Cottrell JN, Cornelius DC, Cunningham MW, Faulkner JL, Ibrahim T, Lamarca B, Amaral LM. 17-Hydroxyprogesterone caproate improves T cells and NK cells in response to placental ischemia; new mechanisms of action for an old drug. Pregnancy Hypertens 19: 226–232, 2020. doi:10.1016/J.PREGHY.2019.11.005. 31806502 PMC7152948

[56] El-Saka MH, Madi NM, Ibrahim RR, Alghazaly GM, Elshwaikh S, El-Bermawy M. The ameliorative effect of angiotensin 1-7 on experimentally induced-preeclampsia in rats: targeting the role of peroxisome proliferator-activated receptors gamma expression & asymmetric dimethylarginine. Arch Biochem Biophys 671: 123–129, 2019. doi:10.1016/J.ABB.2019.07.006. 31295432

[57] Cristóvão Escouto D, Gadonski G, Porcello-Marrone L, Costa da Costa J, Paludo Rr do A N, Costa B. E P D, Poli-de-Figueiredo CE. Using the reduced uterine perfusion pressure model of preeclampsia to study the blood brain barrier permeability. Sci Med 28: 29631, 2018.doi:10.15448/1980-6108.2018.2.29631.

[58] Faulkner JL, Cornelius DC, Amaral LM, Harmon AC, Cunningham MW, Darby MM, Ibrahim T, Thomas DAS, Herse F, Wallukat G, Dechend R, Lamarca B. Vitamin D supplementation improves pathophysiology in a rat model of preeclampsia. Am J Physiol Regul Integr Comp Physiol 310: R346–R354, 2016. doi:10.1152/AJPREGU.00388.2015. 26676250 PMC4796742

[59] Faulkner JL, Plenty NL, Wallace K, Amaral LM, Cunningham MW, Murphy S, LaMarca B. Selective inhibition of 20-hydroxyeicosatetraenoic acid lowers blood pressure in a rat model of preeclampsia. Prostaglandins Other Lipid Mediat 134: 108–113, 2018. doi:10.1016/J.PROSTAGLANDINS.2017.09.004. 28951260 PMC5902033

[60] Fraser GM, Morton JS, Schmidt SM, Bourque S, Davidge ST, Davenport MH, Steinback CD. Reduced uterine perfusion pressure decreases functional capillary density in skeletal muscle. Am J Physiol Heart Circ Physiol 309: H2002–H2007, 2015. doi:10.1152/AJPHEART.00641.2015. 26475590

[61] Gadonski G, LaMarca BBD, Sullivan E, Bennett W, Chandler D, Granger JP. Hypertension produced by reductions in uterine perfusion in the pregnant rat: role of interleukin 6. Hypertension 48: 711–716, 2006. doi:10.1161/01.HYP.0000238442.33463.94. 16940225

[62] George EM, Cockrell K, Aranay M, Csongradi E, Stec DE, Granger JP. Induction of heme oxygenase 1 attenuates placental ischemia-induced hypertension. Hypertension 57: 941–948, 2011. doi:10.1161/HYPERTENSIONAHA.111.169755. 21383306 PMC3085942

[63] George EM, Palei AC, Dent EA, Granger JP. Sildenafil attenuates placental ischemia-induced hypertension. Am J Physiol Regul Integr Comp Physiol 305: R397–R403, 2013. doi:10.1152/AJPREGU.00216.2013. 23785075 PMC3833396

[64] George EM, Garrett MR, Granger JP. Placental ischemia induces changes in gene expression in chorionic tissue. Mamm Genome 25: 253–261, 2014. doi:10.1007/S00335-014-9505-3. 24668059 PMC4238427

[65] Giambrone AB, Logue OC, Shao Q, Bidwell GL 3rd, Warrington JP. Perinatal micro-bleeds and neuroinflammation in E19 rat fetuses exposed to utero-placental ischemia. Int J Mol Sci 20: 4051, 2019. doi:10.3390/IJMS20164051. 31434191 PMC6720786

[66] Giardina JB, Cockrell KL, Granger JP, Khalil RA. Low-salt diet enhances vascular reactivity and Ca(2+) entry in pregnant rats with normal and reduced uterine perfusion pressure. Hypertension 39: 368–374, 2002. doi:10.1161/HY02T2.102806. 11882575

[67] Gilbert J, Dukes M, LaMarca B, Cockrell K, Babcock S, Granger J. Effects of reduced uterine perfusion pressure on blood pressure and metabolic factors in pregnant rats. Am J Hypertens 20: 686–691, 2007. doi:10.1016/J.AMJHYPER.2006.12.016. 17531929

[68] Gilbert JS, Babcock SA, Granger JP. Hypertension produced by reduced uterine perfusion in pregnant rats is associated with increased soluble fms-like tyrosine kinase-1 expression. Hypertension 50: 1142–1147, 2007. doi:10.1161/HYPERTENSIONAHA.107.096594. 17923588

[69] Gilbert JS, Gilbert SAB, Arany M, Granger JP. Hypertension produced by placental ischemia in pregnant rats is associated with increased soluble endoglin expression. Hypertension 53: 399–403, 2009. doi:10.1161/HYPERTENSIONAHA.108.123513. 19075097 PMC2692089

[70] Gilbert JS, Verzwyvelt J, Colson D, Arany M, Karumanchi SA, Granger JP. Recombinant vascular endothelial growth factor 121 infusion lowers blood pressure and improves renal function in rats with placentalischemia-induced hypertension. Hypertension 55: 380–385, 2010. doi:10.1161/HYPERTENSIONAHA.109.141937. 20026764 PMC2824248

[71] Gilbert JS, Bauer AJ, Gingery A, Banek CT, Chasson S. Circulating and utero-placental adaptations to chronic placental ischemia in the rat. Placenta 33: 100–105, 2012. doi:10.1016/J.PLACENTA.2011.11.025. 22185915

[72] Gutkowska J, Granger JP, Lamarca BB, Danalache BA, Wang D, Jankowski M. Changes in cardiac structure in hypertension produced by placental ischemia in pregnant rats: effect of tumor necrosis factor blockade. J Hypertens 29: 1203–1212, 2011. doi:10.1097/HJH.0B013E3283468392. 21505354

[73] Haase N, Foster DJ, Cunningham MW, Bercher J, Nguyen T, Shulga-Morskaya S, Milstein S, Shaikh S, Rollins J, Golic M, Herse F, Kräker K, Bendix I, Serdar M, Napieczynska H, Heuser A, Gellhaus A, Thiele K, Wallukat G, Müller DN, LaMarca B, Dechend R. RNA interference therapeutics targeting angiotensinogen ameliorate preeclamptic phenotype in rodent models. J Clin Invest 130: 2928–2942, 2020. doi:10.1172/JCI99417. 32338644 PMC7260005

[74] Han N, Li Y, Dong Y. Therapeutic effect of long-term epidural block in rats with pregnancy induced hypertension. Biomed Res Int 2018: 1639623, 2018. doi:10.1155/2018/1639623. 29568742 PMC5820560

[75] Harmon A, Cornelius D, Amaral L, Paige A, Herse F, Ibrahim T, Wallukat G, Faulkner J, Moseley J, Dechend R, LaMarca B. IL-10 supplementation increases Tregs and decreases hypertension in the RUPP rat model of preeclampsia. Hypertens Pregnancy 34: 291–306, 2015. doi:10.3109/10641955.2015.1032054. 25996051 PMC4904776

[76] Hassanzadeh-Taheri M, Mohammadifard M, Erfanian Z, Hosseini M. The maternal reduced uteroplacental perfusion model of preeclampsia induces sexually dimorphic metabolic responses in rat offspring. Biol Sex Differ 13: 48, 2022. doi:10.1186/S13293-022-00458-8. 36109770 PMC9479437

[77] Heltemes A, Gingery A, Soldner ELB, Bozadjieva N, Jahr KN, Johnson BK, Gilbert JS. Chronic placental ischemia alters amniotic fluid milieu and results in impaired glucose tolerance, insulin resistance and hyperleptinemia in young rats. Exp Biol Med (Maywood) 235: 892–899, 2010. doi:10.1258/EBM.2010.009357. 20558843

[78] Herrock O, Deer E, Amaral LM, Campbell N, Whitney D, Ingram N, Cornelius DC, Turner T, Hardy-Hardin J, Booz GW, Ibrahim T, LaMarca B. Inhibiting B cell activating factor attenuates preeclamptic symptoms in placental ischemic rats. Am J Reprod Immunol 89: e13693, 2023. doi:10.1111/AJI.13693. 36794639 PMC10009902

[79] Herrock OT, Deer E, Amaral LM, Campbell N, Lemon J, Ingram N, Cornelius DC, Turner TW, Fitzgerald S, Ibrahim T, Dechend R, Wallukat G, LaMarca B. B2 cells contribute to hypertension and natural killer cell activation possibly via AT1-AA in response to placental ischemia. Am J Physiol Renal Physiol 324: F179–F192, 2023. doi:10.1152/AJPRENAL.00190.2022. 36417275 PMC9844978

[80] Herse F, Lamarca B, Hubel CA, Kaartokallio T, Lokki AI, Ekholm E, Laivuori H, Gauster M, Huppertz B, Sugulle M, Ryan MJ, Novotny S, Brewer J, Park JK, Kacik M, Hoyer J, Verlohren S, Wallukat G, Rothe M, Luft FC, Muller DN, Schunck WH, Staff AC, Dechend R. Cytochrome P450 subfamily 2J polypeptide 2 expression and circulating epoxyeicosatrienoic metabolites in preeclampsia. Circulation 126: 2990–2999, 2012. doi:10.1161/CIRCULATIONAHA.112.127340. 23155181 PMC3543781

[81] Hines T, Beauchamp D, Rice C. Baroreflex control of sympathetic nerve activity in hypertensive pregnant rats with reduced uterine perfusion. Hypertens Pregnancy 26: 303–314, 2007. doi:10.1080/10641950701415598. 17710579

[82] Huang Y, Zheng XD, Li H. Protective role of SIRT1-mediated sonic Hedgehog signaling pathway in the preeclampsia rat models. J Assist Reprod Genet 38: 1843–1851, 2021. doi:10.1007/S10815-021-02158-5. 33772412 PMC8324598

[83] Ibrahim T, Przybyl L, Harmon AC, Amaral LM, Faulkner JL, Cornelius DC, Cunningham MW, Hünig T, Herse F, Wallukat G, Dechend R, LaMarca B. Proliferation of endogenous regulatory T cells improve the pathophysiology associated with placental ischaemia of pregnancy. Am J Rep Immunol. 6 July 2017 [Epub ahead of print]. doi:10.1111/AJI.12724. 28681467 PMC5916789

[84] Intapad S, Warrington JP, Spradley FT, Palei AC, Drummond HA, Ryan MJ, Granger JP, Alexander BT. Reduced uterine perfusion pressure induces hypertension in the pregnant mouse. Am J Physiol Regul Integr Comp Physiol 307: R1353–R1357, 2014. doi:10.1152/AJPREGU.00268.2014. 25298513 PMC4254941

[85] Isler CM, Bennett WA, Rinewalt AN, Cockrell KL, Martin JN, Morrison JC, Granger JP. Evaluation of a rat model of preeclampsia for HELLP syndrome characteristics. J Soc Gynecol Investig 10: 151–153, 2003. doi:10.1016/s1071-5576(03)00009-1. 12699877

[86] Issotina Zibrila A, Li Y, Wang Z, Zhao G, Liu H, Leng J, Ahasan Ali M, Ampofo Osei J, Kang YM, Liu J. Acetylcholinesterase inhibition with pyridostigmine attenuates hypertension and neuroinflammation in the paraventricular nucleus in rat model for preeclampsia. Int Immunopharmacol 101: 108365, 2021. doi:10.1016/J.INTIMP.2021.108365. 34815190

[87] Issotina Zibrila A, Wang Z, Ali MA, Osei JA, Sun Y, Zafar S, Liu K, Li C, Kang Y, Liu J. Pyridostigmine ameliorates preeclamptic features in pregnant rats by inhibiting tumour necrosis factor-α synthetsis and antagonizing tumour necrosis factor-α-related effects. J Hypertens 39: 1774–1789, 2021. doi:10.1097/HJH.0000000000002932. 34232157

[88] Javadian P, Salmanian B, Javadi-Paydar M, Shamshirsaz AA, Ejtemaei Mehr S, Gharedaghi MH, Dehpour AR. Effect of morphine on the reduced uteroplacental perfusion model of pre-eclampsia in rats. Eur J Obstet Gynecol Reprod Biol 168: 161–166, 2013. doi:10.1016/J.EJOGRB.2013.01.008. 23398725

[89] Joyner J, Neves LAA, Granger JP, Alexander BT, Merrill DC, Chappell MC, Ferrario CM, Davis WP, Brosnihan KB. Temporal-spatial expression of ANG-(1-7) and angiotensin-converting enzyme 2 in the kidney of normal and hypertensive pregnant rats. Am J Physiol Regul Integr Comp Physiol 293: R169–R177, 2007. doi:10.1152/AJPREGU.00387.2006. 17428896

[90] Kiprono LV, Wallace K, Moseley J, Martin J, LaMarca B. Progesterone blunts vascular endothelial cell secretion of endothelin-1 in response to placental ischemia. Am J Obstet Gynecol 209: 44.e1–44.e6, 2013. doi:10.1016/J.AJOG.2013.03.032. 23545163 PMC4052216

[91] LaMarca BBD, Bennett WA, Alexander BT, Cockrell K, Granger JP. Hypertension produced by reductions in uterine perfusion in the pregnant rat: role of tumor necrosis factor-α. Hypertension 46: 1022–1025, 2005. doi:10.1161/01.HYP.0000175476.26719.36. 16144982

[92] LaMarca B, Wallukat G, Llinas M, Herse F, Dechend R, Granger JP. Autoantibodies to the angiotensin type I receptor in response to placental ischemia and tumor necrosis factor α in pregnant rats. Hypertension 52: 1168–1172, 2008. doi:10.1161/HYPERTENSIONAHA.108.120576. 18852381 PMC2782765

[93] LaMarca B, Speed J, Fournier L, Babcock SA, Berry H, Cockrell K, Granger JP. Hypertension in response to chronic reductions in uterine perfusion in pregnant rats. Hypertension 52: 1161–1167, 2008. doi:10.1161/HYPERTENSIONAHA.108.120881. 18981324 PMC2788766

[94] Lamarca B, Wallace K, Herse F, Wallukat G, Martin JN, Weimer A, Dechend R. Hypertension in response to placental ischemia during pregnancy: role of B lymphocytes. Hypertension 57: 865–871, 2011. doi:10.1161/HYPERTENSIONAHA.110.167569. 21357287 PMC3109629

[95] Laule CF, Wing CR, Odean EJ, Wilcox JA, Gilbert JS, Regal JF. Effect of nicotine on placental ischemia-induced complement activation and hypertension in the rat. J Immunotoxicol 14: 235–240, 2017. doi:10.1080/1547691X.2017.1394934. 29185370 PMC6298215

[96] Laule CF, Odean EJ, Wing CR, Root KM, Towner KJ, Hamm CM, Gilbert JS, Fleming SD, Regal JF. Role of B1 and B2 lymphocytes in placental ischemia-induced hypertension. Am J Physiol Heart Circ Physiol 317: H732–H742, 2019. doi:10.1152/AJPHEART.00132.2019. 31397167 PMC6843018

[97] Lawrence DJ, Escott ME, Myers L, Intapad S, Lindsey SH, Bayer CL. Spectral photoacoustic imaging to estimate in vivo placental oxygenation during preeclampsia. Sci Rep 9: 558, 2019. doi:10.1038/S41598-018-37310-2. 30679723 PMC6345947

[98] Lawrence DJ, Bayer CL. Photoacoustic imaging provides an in vivo assessment of the preeclamptic placenta remodeling and function in response to therapy. Placenta 126: 46–53, 2022. doi:10.1016/J.PLACENTA.2022.06.006. 35764022 PMC10236486

[99] Li W, Cui N, Mazzuca MQ, Mata KM, Khalil RA. Increased vascular and uteroplacental matrix metalloproteinase-1 and -7 levels and collagen type I deposition in hypertension in pregnancy: role of TNF-α. Am J Physiol Heart Circ Physiol 313: H491–H507, 2017. doi:10.1152/AJPHEART.00207.2017. 28626073 PMC5625170

[100] Lillegard KE, Johnson AC, Lojovich SJ, Bauer AJ, Marsh HC, Gilbert JS, Regal JF. Complement activation is critical for placental ischemia-induced hypertension in the rat. Mol Immunol 56: 91–97, 2013. doi:10.1016/J.MOLIMM.2013.04.009. 23685261 PMC3687024

[101] Lillegard KE, Loeks-Johnson AC, Opacich JW, Peterson JM, Bauer AJ, Elmquist BJ, Regal RR, Gilbert JS, Regal JF. Differential effects of complement activation products c3a and c5a on cardiovascular function in hypertensive pregnant rats. J Pharmacol Exp Ther 351: 344–351, 2014. doi:10.1124/JPET.114.218123. 25150279 PMC4201271

[102] Lin C, He H, Cui N, Ren Z, Zhu M, Khalil RA. Decreased uterine vascularization and uterine arterial expansive remodeling with reduced matrix metalloproteinase-2 and -9 in hypertensive pregnancy. Am J Physiol Heart Circ Physiol 318: H165–H180, 2020. doi:10.1152/AJPHEART.00602.2019. 31834839 PMC6985805

[103] Liu L, Wang R, Xu R, Chu Y, Gu W. Procyanidin B2 ameliorates endothelial dysfunction and impaired angiogenesis via the Nrf2/PPARγ/sFlt-1 axis in preeclampsia. Pharmacol Res 177: 106127, 2022. doi:10.1016/J.PHRS.2022.106127. 35150862

[104] Llinás MT, Alexander BT, Seedek M, Abram SR, Crell A, Granger JP. Enhanced thromboxane synthesis during chronic reductions in uterine perfusion pressure in pregnant rats. Am J Hypertens 15: 793–797, 2002. doi:10.1016/S0895-7061(02)02975-8. 12219874

[105] Logue OC, Mahdi F, Chapman H, George EM, Bidwell GL. A maternally sequestered, biopolymer-stabilized vascular endothelial growth factor (VEGF) chimera for treatment of preeclampsia. J Am Heart Assoc 6: e007216, 2017. doi:10.1161/JAHA.117.007216. 29629873 PMC5779036

[106] Ma SL, Tian XY, Wang YQ, Zhang HF, Zhang L. Vitamin D supplementation prevents placental ischemia induced endothelial dysfunction by downregulating placental soluble FMS-like tyrosine kinase-1. DNA Cell Biol 36: 1134–1141, 2017. doi:10.1089/DNA.2017.3817. 28981319

[107] Porcello Marrone LC, Gadonski G, De Oliveira Laguna G, Poli-De-Figueiredo CE, Pinheiro Da Costa BE, Lopes MFT, Brunelli JPF, Diogo LP, Huf Marrone AC, Da Costa JC. Blood-brain barrier breakdown in reduced uterine perfusion pressure: a possible model of posterior reversible encephalopathy syndrome. J Stroke Cerebrovasc Dis 23: 2075–2079, 2014. doi:10.1016/J.JSTROKECEREBROVASDIS.2014.03.012. 25113078

[108] Mazzuca MQ, Li W, Reslan OM, Yu P, Mata KM, Khalil RA. Downregulation of microvascular endothelial type B endothelin receptor is a central vascular mechanism in hypertensive pregnancy. Hypertension 64: 632–643, 2014. doi:10.1161/HYPERTENSIONAHA.114.03315. 24914193 PMC4134432

[109] Mazzuca MQ, Buyukcelebi K, Lin C, Khalil RA. Increased Ca^2+^-dependent intrinsic tone and arterial stiffness in mesenteric microvessels of hypertensive pregnant rats. Biochem Pharmacol 208: 115353, 2023. doi:10.1016/J.BCP.2022.115353. 36435203 PMC9877182

[110] McCarthy FP, Drewlo S, Kingdom J, Johns EJ, Walsh SK, Kenny LC. Peroxisome proliferator-activated receptor-γ as a potential therapeutic target in the treatment of preeclampsia. Hypertension 58: 280–286, 2011. doi:10.1161/HYPERTENSIONAHA.111.172627. 21690483

[111] Moore LE, Wallace KL, Alexander BT, May WL, Thigpen BD, Bennett WA. Reduced placental perfusion causes an increase in maternal serum leptin. Placenta 24: 877–881, 2003. doi:10.1016/S0143-4004(03)00139-5. 13129685

[112] Morton JS, Abdalvand A, Jiang Y, Sawamura T, Uwiera RRE, Davidge ST. Lectin-like oxidized low-density lipoprotein 1 receptor in a reduced uteroplacental perfusion pressure rat model of preeclampsia. Hypertension 59: 1014–1020, 2012. doi:10.1161/HYPERTENSIONAHA.112.191825. 22392901

[113] Morton JS, Quon A, Cheung PY, Sawamura T, Davidge ST. Effect of sodium tanshinone IIA sulfonate treatment in a rat model of preeclampsia. Am J Physiol Regul Integr Comp Physiol 308: R163–R172, 2015. doi:10.1152/AJPREGU.00222.2014. 25477421

[114] Morton JS, Levasseur J, Ganguly E, Quon A, Kirschenman R, Dyck JRB, Fraser GM, Davidge ST. Characterisation of the selective reduced uteroplacental perfusion (sRUPP) model of preeclampsia. Sci Rep 9: 9565, 2019. doi:10.1038/S41598-019-45959-6. 31266978 PMC6606748

[115] Murphy SR, Cockrell K. Regulation of soluble fms-like tyrosine kinase-1 production in response to placental ischemia/hypoxia: role of angiotensin II. Physiol Rep 3: e12310, 2015. doi:10.14814/PHY2.12310. 25716926 PMC4393214

[116] Neves LAA, Stovall K, Joyner JN, Valdés G, Gallagher PE, Ferrario CM, Merrill DC, Brosnihan KB. ACE2 and ANG-(1-7) in the rat uterus during early and late gestation. Am J Physiol Regul Integr Comp Physiol 294: R151–R161, 2008. doi:10.1152/AJPREGU.00514.2007. 17977916

[117] Novotny SR, Wallace K, Heath J, Moseley J, Dhillon P, Weimer A, Wallukat G, Herse F, Wenzel K, Martin JN, Dechend R, LaMarca B. Activating autoantibodies to the angiotensin II type I receptor play an important role in mediating hypertension in response to adoptive transfer of CD4+ T lymphocytes from placental ischemic rats. Am J Physiol Regul Integr Comp Physiol 302: R1197–R1201, 2012. doi:10.1152/AJPREGU.00623.2011. 22461177 PMC3362148

[118] Novotny S, Wallace K, Herse F, Moseley J, Darby M, Heath J, Gill J, Wallukat G, Martin JN, Dechend R, LaMarca B. CD4+ T cells play a critical role in mediating hypertension in response to placental ischemia. J Hypertens (Los Angel) 2: 14873, 2013. doi:10.4172/2167-1095.1000116. 25401050 PMC4231445

[119] Ojeda N, Hall S, Lasley CJ, Rudsenske B, Dixit M, Arany I.. Prenatal nicotine exposure augments renal oxidative stress in embryos of pregnant rats with reduced uterine perfusion pressure In Vivo 30: 219–224, 2016. 27107078

[120] Ou M, Zhao H, Ji G, Zhao X, Zhang Q. Long noncoding RNA MALAT1 contributes to pregnancy-induced hypertension development by enhancing oxidative stress and inflammation through the regulation of the miR-150-5p/ET-1 axis. FASEB J 34: 6070–6085, 2020. doi:10.1096/FJ.201902280R. 32246794

[121] Paauw ND, Joles JA, Spradley FT, Bakrania B, Zsengeller ZK, Franx A, Verhaar MC, Granger JP, Lely AT. Exposure to placental ischemia impairs postpartum maternal renal and cardiac function in rats. Am J Physiol Regul Integr Comp Physiol 312: R664–R670, 2017. doi:10.1152/AJPREGU.00510.2016. 28202440 PMC5451567

[122] Palei AC, Martin HL, Wilson BA, Anderson CD, Granger JP, Spradley FT. Impact of hyperleptinemia during placental ischemia-induced hypertension in pregnant rats. Am J Physiol Heart Circ Physiol 320: H1949–H1958, 2021. doi:10.1152/AJPHEART.00724.2019. 33710923 PMC8163645

[123] Pang H, Lei D, Huang J, Guo Y, Fan C. Elevated PGT promotes proliferation and inhibits cell apoptosis in preeclampsia by Erk signaling pathway. Mol Cell Probes 67: 101896, 2023. doi:10.1016/J.MCP.2023.101896. 36731680

[124] Ramirez RJJ, Debrah J, Novak J. Increased myogenic responses of resistance-sized mesenteric arteries after reduced uterine perfusion pressure in pregnant rats. Hypertens Pregnancy 30: 45–57, 2011. doi:10.3109/10641950903322923. 20818955

[125] Regal JF, Strehlke ME, Peterson JM, Wing CR, Parker JE, Nieto NF, Bemis LT, Gilbert JS, Fleming SD. Role of IgM and angiotensin II Type I receptor autoantibodies in local complement activation in placental ischemia-induced hypertension in the rat. Mol Immunol 78: 38–47, 2016. doi:10.1016/J.MOLIMM.2016.08.016. 27588825 PMC5056842

[126] Regal JF, Lund JM, Wing CR, Root KM, McCutcheon L, Bemis LT, Gilbert JS, Fleming SD. Interactions between the complement and endothelin systems in normal pregnancy and following placental ischemia. Mol Immunol 114: 10–18, 2019. doi:10.1016/J.MOLIMM.2019.06.015. 31326653 PMC6774867

[127] Reho JJ, Peck J, Novak J, Ramirez RJ. Hypertension induced by episodic reductions in uteroplacental blood flow in gravid rat. Hypertens Pregnancy 30: 208–220, 2011. doi:10.3109/10641955.2010.507853. 20846047

[128] Ren Z, Cui N, Zhu M, Khalil RA. Placental growth factor reverses decreased vascular and uteroplacental MMP-2 and MMP-9 and increased MMP-1 and MMP-7 and collagen types I and IV in hypertensive pregnancy. Am J Physiol Heart Circ Physiol 315: H33–H47, 2018. doi:10.1152/AJPHEART.00045.2018. 29569955 PMC6087780

[129] Ren Z, Cui N, Zhu M, Khalil RA. TNFα blockade reverses vascular and uteroplacental matrix metalloproteinases imbalance and collagen accumulation in hypertensive pregnant rats. Biochem Pharmacol 193: 114790, 2021. doi:10.1016/J.BCP.2021.114790. 34600915 PMC8606001

[130] Ryan MJ, Gilbert EL, Glover PH, George EM, Warren Masterson C, McLemore GR, Lamarca B, Granger JP, Drummond HA. Placental ischemia impairs middle cerebral artery myogenic responses in the pregnant rat. Hypertension 58: 1126–1131, 2011. doi:10.1161/HYPERTENSIONAHA.111.181453. 22068864 PMC3488858

[131] Santiago-Font JA, Amaral LM, Faulkner J, Ibrahim T, Vaka VR, Cunningham MW, Lamarca B. Serelaxin improves the pathophysiology of placental ischemia in the reduced uterine perfusion pressure rat model of preeclampsia. Am J Physiol Regul Integr Comp Physiol 311: R1158–R1163, 2016. doi:10.1152/AJPREGU.00192.2016. 27629886 PMC5256981

[132] Sedeek M, Gilbert JS, Lamarca BB, Sholook M, Chandler DL, Wang Y, Granger JP. Role of reactive oxygen species in hypertension produced by reduced uterine perfusion in pregnant rats. Am J Hypertens 21: 1152–1156, 2008. doi:10.1038/AJH.2008.239. 18670418 PMC2786058

[133] Sholook MM, Gilbert JS, Sedeek MH, Huang M, Hester RL, Granger JP. Systemic hemodynamic and regional blood flow changes in response to chronic reductions in uterine perfusion pressure in pregnant rats. Am J Physiol Heart Circ Physiol 293: H2080–H2084, 2007. doi:10.1152/AJPHEART.00667.2007. 17644567

[134] Spradley FT, Tan AY, Joo WS, Daniels G, Kussie P, Karumanchi SA, Granger JP. Placental growth factor administration abolishes placental ischemia-induced hypertension. Hypertension 67: 740–747, 2016. doi:10.1161/HYPERTENSIONAHA.115.06783. 26831193 PMC4786447

[135] Spradley FT, Ge Y, Haynes BP, Granger JP, Anderson CD. Adrenergic receptor blockade attenuates placental ischemia-induced hypertension. Physiol Rep 6: e13814, 2018. doi:10.14814/PHY2.13814. 30229567 PMC6121121

[136] Spradley FT, Palei AC, Anderson CD, Granger JP. Melanocortin-4 receptor deficiency attenuates placental ischemia-induced hypertension in pregnant rats. Hypertension 73: 162–170, 2019. doi:10.1161/HYPERTENSIONAHA.118.12028. 30571561 PMC6309903

[137] Sun X, Zhang S, Song H. Quercetin attenuates reduced uterine perfusion pressure -induced hypertension in pregnant rats through regulation of endothelin-1 and endothelin-1 type A receptor. Lipids Health Dis 19: 180, 2020. doi:10.1186/S12944-020-01357-W. 32758232 PMC7409636

[138] Tam KB, George E, Cockrell K, Arany M, Speed J, Martin JN, Lamarca B, Granger JP. Endothelin type A receptor antagonist attenuates placental ischemia-induced hypertension and uterine vascular resistance. Am J Obstet Gynecol 204: 330.e1–330.e4, 2011. doi:10.1016/J.AJOG.2011.01.049. 21458623 PMC3072697

[139] Tian X, Ma S, Wang Y, Hou L, Shi Y, Yao M, Wang X, Zhang H, Jiang L. Effects of placental ischemia are attenuated by 1,25-dihydroxyvitamin D treatment and associated with reduced apoptosis and increased autophagy. DNA Cell Biol 35: 59–70, 2016. doi:10.1089/DNA.2015.2885. 26562100

[140] Travis OK, White D, Baik C, Giachelli C, Thompson W, Stubbs C, Greer M, Lemon JP, Williams JM, Cornelius DC. Interleukin-17 signaling mediates cytolytic natural killer cell activation in response to placental ischemia. Am J Physiol Regul Integr Comp Physiol 318: R1036–R1046, 2020. doi:10.1152/AJPREGU.00285.2019. 32320265 PMC7311683

[141] Travis OK, Baik C, Tardo GA, Amaral L, Jackson C, Greer M, Giachelli C, Ibrahim T, Herrock OT, Williams JM, Cornelius DC. Adoptive transfer of placental ischemia-stimulated natural killer cells causes a preeclampsia-like phenotype in pregnant rats. Am J Reprod Immunol 85: e13386, 2021. doi:10.1111/AJI.13386. 33315281 PMC8131208

[142] Travis OK, Tardo GA, Giachelli C, Siddiq S, Nguyen HT, Crosby MT, Johnson TD, Brown AK, Booz GW, Smith AN, Williams JM, Cornelius DC. Interferon c neutralization reduces blood pressure, uterine artery resistance index, and placental oxidative stress in placental ischemic rats. Am J Physiol Regul Integr Comp Physiol 321: R112–R124, 2021. doi:10.1152/AJPREGU.00349.2020. 34075808 PMC8409917

[143] Travis OK, Tardo GA, Giachelli C, Siddiq S, Nguyen HT, Crosby MT, Johnson T, Brown AK, Williams JM, Cornelius DC. Tumor necrosis factor- α blockade improves uterine artery resistance, maternal blood pressure, and fetal growth in placental ischemic rats. Pregnancy Hypertens 25: 39–47, 2021. doi:10.1016/J.PREGHY.2021.05.002. 34051437 PMC8363517

[144] Ushida T, Kotani T, Tsuda H, Imai K, Nakano T, Hirako S, Ito Y, Li H, Mano Y, Wang J, Miki R, Yamamoto E, Iwase A, Bando YK, Hirayama M, Ohno K, Toyokuni S, Kikkawa F. Molecular hydrogen ameliorates several characteristics of preeclampsia in the reduced uterine perfusion pressure (RUPP) rat model. Free Radic Biol Med 101: 524–533, 2016. doi:10.1016/J.FREERADBIOMED.2016.10.491. 27789293

[145] Vaka VR, McMaster KM, Cunningham MW, Ibrahim T, Hazlewood R, Usry N, Cornelius DC, Amaral LM, LaMarca B. Role of mitochondrial dysfunction and reactive oxygen species in mediating hypertension in the reduced uterine perfusion pressure rat model of preeclampsia. Hypertension 72: 703–711, 2018. doi:10.1161/HYPERTENSIONAHA.118.11290. 30012871 PMC6394841

[146] Vaka VR, McMaster KM, Cornelius DC, Ibrahim T, Jayaram A, Usry N, Cunningham MW, Amaral LM, LaMarca B. Natural killer cells contribute to mitochondrial dysfunction in response to placental ischemia in reduced uterine perfusion pressure rats. Am J Physiol Regul Integr Comp Physiol 316: R441–R447, 2019. doi:10.1152/AJPREGU.00279.2018. 30811248 PMC6589603

[147] Veillon EW, Keiser SD, Parrish MR, Bennett W, Cockrell K, Ray LF, Granger JP, Martin JN, LaMarca B. 17-Hydroxyprogesterone blunts the hypertensive response associated with reductions in uterine perfusion pressure in pregnant rats. Am J Obstet Gynecol 201: 324.e1–324.e6, 2009. doi:10.1016/J.AJOG.2009.05.054. 19733289 PMC2810642

[148] Walsh SK, English FA, Johns EJ, Kenny LC. Plasma-mediated vascular dysfunction in the reduced uterine perfusion pressure model of preeclampsia: a microvascular characterization. Hypertension 54: 345–351, 2009. doi:10.1161/HYPERTENSIONAHA.109.132191. 19564546

[149] Walsh SK, English FA, Crocker IP, Johns EJ, Kenny LC. Contribution of PARP to endothelial dysfunction and hypertension in a rat model of pre-eclampsia. Br J Pharmacol 166: 2109–2116, 2012. doi:10.1111/J.1476-5381.2012.01906.X. 22339234 PMC3402775

[150] Wang X, Travis OK, Shields CA, Tardo GA, Giachelli C, Nutter CW, Glenn HL, Cooper OG, Davis T, Thomas R, Williams JM, Cornelius DC. NLRP3 inhibition improves maternal hypertension, inflammation, and vascular dysfunction in response to placental ischemia. Am J Physiol Regul Integr Comp Physiol 324: R556–R567, 2023. doi:10.1152/AJPREGU.00192.2022. 36847598 PMC10069976

[151] Warrington JP, Drummond HA, Granger JP, Ryan MJ. Placental ischemia-induced increases in brain water content and cerebrovascular permeability: role of TNF-α. Am J Physiol Regul Integr Comp Physiol 309: R1425–R1431, 2015. doi:10.1152/AJPREGU.00372.2015. 26400187 PMC4698405

[152] Li W, Mata KM, Mazzuca MQ, Khalil RA. Altered matrix metalloproteinase-2 and -9 expression/activity links placental ischemia and anti-angiogenic sFlt-1 to uteroplacental and vascular remodeling and collagen deposition in hypertensive pregnancy. Biochem Pharmacol 89: 370–385, 2014. doi:10.1016/J.BCP.2014.03.017. 24704473 PMC4034157

[153] Wei J, Zhang J, Jiang S, Xu L, Qu L, Pang B, Jiang K, Wang L, Intapad S, Buggs J, Cheng F, Mohapatra S, Juncos LA, Osborn JL, Granger JP, Liu R. Macula densa NOS1β modulates renal hemodynamics and blood pressure during pregnancy: role in gestational hypertension. J Am Soc Nephrol 32: 2485–2500, 2021. doi:10.1681/ASN.2020070969. 34127535 PMC8722793

[154] Williamson RD, McCarthy FP, Manna S, Groarke E, Kell DB, Kenny LC, McCarthy CM. L-(+)-Ergothioneine significantly improves the clinical characteristics of preeclampsia in the reduced uterine perfusion pressure rat model. Hypertension 75: 561–568, 2020. doi:10.1161/HYPERTENSIONAHA.119.13929. 31865793

[155] Yang Y, Li JP, Bian Y, Song GY, Li YY, Zheng DY, Huang L, Qiao C. Lin28B expression in reduced uterine perfusion pressure rat model. Reprod Develop Med 3: 36–41, 2019. doi:10.4103/2096-2924.255987.

[156] Yang Z, Jia X, Deng Q, Luo M, Hou Y, Yue J, Mei J, Wu Z, Shan N. Human umbilical cord mesenchymal stem cell-derived extracellular vesicles loaded with TFCP2 activate Wnt/β-catenin signaling to alleviate preeclampsia. Int Immunopharmacol 115: 109732, 2023. doi:10.1016/J.INTIMP.2023.109732. 37724958

[157] Younes ST, Maeda KJ, Sasser J, Ryan MJ. The glucagon-like peptide 1 receptor agonist liraglutide attenuates placental ischemia-induced hypertension. Am J Physiol Heart Circ Physiol 318: H72–H77, 2020. doi:10.1152/AJPHEART.00486.2019. 31729903 PMC6985807

[158] Zhang LW, Warrington JP. Magnesium sulfate prevents placental ischemia-induced increases in brain water content and cerebrospinal fluid cytokines in pregnant rats. Front Neurosci 10: 561, 2016. doi:10.3389/FNINS.2016.00561. 28008305 PMC5143678

[159] Zhang H, He Y, Wang JX, Chen MH, Xu JJ, Jiang MH, Feng YL, Ling Gu YF. miR-30-5p-mediated ferroptosis of trophoblasts is implicated in the pathogenesis of preeclampsia. Redox Biol 29: 101402, 2020. doi:10.1016/J.REDOX.2019.101402. 31926626 PMC6928320

[160] Zhang T, Guo D, Zheng W, Dai Q. Effects of S1PR2 antagonist on blood pressure and angiogenesis imbalance in preeclampsia rats. Mol Med Rep 23: 456, 2021. doi:10.3892/MMR.2021.12095. 33880585

[161] Zhang ML, Yang Q, Zhu YD, Zhang YD, Zhang R, Liu J, Zhao XY, Dang QY, Huang DX, Zhang MY, Wei YC, Hu Z, Cai XX, Gao LF, Shan Y, Yu HL. Nobiletin Inhibits Hypoxia-Induced Placental Damage via Modulating P53 Signaling Pathway. Nutrients 14: 2332, 2022. doi:10.3390/NU14112332. 35684132 PMC9183106

[162] Zheng H, Yu Z, Wang H, Liu H, Chen X. MiR-125b-5p ameliorates hypoxia/reoxygenation-induced endothelial cell dysfunction and attenuates reduced uterine perfusion pressure-induced hypertension in pregnant rats via targeting BMF. Hypertens Pregnancy 41: 79–88, 2022. doi:10.1080/10641955.2022.2036753. 35171055

[163] Bogdan S, Luca V, Ober C, Melega I, Pestean C, Codea R, Oana L. Comparison among different methods for blood pressure monitoring in rats: literature review. Bull Univ Agricult Sci Vet Med Cluj Napoca Vet Med 76: 5–19, 2019. doi:10.15835/buasvmcn-vm:2019.0007.

[164] Ibrahim J, Berk BC, Hughes AD. Comparison of simultaneous measurements of blood pressure by tail-cuff and carotid arterial methods in conscious spontaneously hypertensive and Wistar-Kyoto rats. Clin Exp Hypertens 28: 57–72, 2006. doi:10.1080/10641960500386817. 16443565

[165] Whitesall SE, Hoff JB, Vollmer AP, D'Alecy LG. Comparison of simultaneous measurement of mouse systolic arterial blood pressure by radiotelemetry and tail-cuff methods. Am J Physiol Heart Circ Physiol 286: H2408–H2415, 2004. doi:10.1152/AJPHEART.01089.2003. 14962829

[166] Olazabal AE, Morrison WA, O'Brien BM. Immediate and short-term effect on arterial flow of clamping or stripping one vessel of a two vessel limb in a dog model. Microsurgery 15: 722–725, 1994. doi:10.1002/MICR.1920151011. 7885219

[167] Ghodsi SR, Esfahanian V, Shamsodini R, Ghodsi SM, Ahmadi G. Blood flow vectoring control in aortic arch using full and partial clamps. Comput Biol Med 43: 1134–1141, 2013. doi:10.1016/J.COMPBIOMED.2013.05.016. 23930806

[168] Bencze M, Behuliak M, Zicha J. The impact of four different classes of anesthetics on the mechanisms of blood pressure regulation in normotensive and spontaneously hypertensive rats. Physiol Res 62: 471–478, 2013. doi:10.33549/PHYSIOLRES.932637. 24020816

[169] Aoi W, Gable D, Cleary RE, Young PCM, Weinberger MH. The antihypertensive effect of pregnancy in spontaneously hypertensive rats. Proc Soc Exp Biol Med 153: 13–15, 1976. doi:10.3181/00379727-153-39471. 995934

[170] Gillis EE, Williams JM, Garrett MR, Mooney JN, Sasser JM. The Dahl salt-sensitive rat is a spontaneous model of superimposed preeclampsia. Am J Physiol Regul Integr Comp Physiol 309: R62–R70, 2015. doi:10.1152/AJPREGU.00377.2014. 25904684 PMC4491533

[171] Deng A, Engels K, Baylis C. Impact of nitric oxide deficiency on blood pressure and glomerular hemodynamic adaptations to pregnancy in the rat. Kidney Int 50: 1132–1138, 1996. doi:10.1038/KI.1996.420. 8887270

[172] Iacono A, Bianco G, Mattace Raso G, Esposito E, d'Emmanuele di Villa Bianca R, Sorrentino R, Cuzzocrea S, Calignano A, Autore G, Meli R. Maternal adaptation in pregnant hypertensive rats: improvement of vascular and inflammatory variables and oxidative damage in the kidney. Am J Hypertens 22: 777–783, 2009. doi:10.1038/AJH.2009.68. 19373215

[173] Sasser JM, Baylis C. Effects of sildenafil on maternal hemodynamics and fetal growth in normal rat pregnancy. Am J Physiol Regul Integr Comp Physiol 298: R433–R438, 2010. doi:10.1152/AJPREGU.00198.2009. 19955496 PMC2828177

[174] Turanov AA, Lo A, Hassler MR, Makris A, Ashar-Patel A, Alterman JF, Coles AH, Haraszti RA, Roux L, Godinho BMDC, Echeverria D, Pears S, Iliopoulos J, Shanmugalingam R, Ogle R, Zsengeller ZK, Hennessy A, Karumanchi SA, Moore MJ, Khvorova A. RNAi modulation of placental sFLT1 for the treatment of preeclampsia. Nat Biotechnol 36: 1164–1173, 2018. doi:10.1038/NBT.4297. 30451990 PMC6526074

[175] Makris A, Thornton C, Thompson J, Thomson S, Martin R, Ogle R, Waugh R, Mckenzie P, Kirwan P, Hennessy A. Uteroplacental ischemia results in proteinuric hypertension and elevated sFLT-1. Kidney Int 71: 977–984, 2007. doi:10.1038/sj.ki.5002175. 17377512

[176] Zhou CC, Ahmad S, Mi T, Abbasi S, Xia L, Day MC, Ramin SM, Ahmed A, Kellems RE, Xia Y. Autoantibody from women with preeclampsia induces soluble Fms-like tyrosine kinase-1 production via angiotensin type 1 receptor and calcineurin/nuclear factor of activated T-cells signaling. Hypertension 51: 1010–1019, 2008. doi:10.1161/HYPERTENSIONAHA.107.097790. 18259044 PMC3261612

[177] Major HD, Campbell RA, Silver RM, Branch DW, Weyrich AS. Synthesis of sFlt-1 by platelet-monocyte aggregates contributes to the pathogenesis of preeclampsia. Am J Obstet Gynecol 210: 547.e1–547.e7, 2014. doi:10.1016/J.AJOG.2014.01.024. 24440566 PMC4041388

[178] Parrish MR, Murphy SR, Rutland S, Wallace K, Wenzel K, Wallukat G, Keiser S, Ray LF, Dechend R, Martin JN, Granger JP, Lamarca B. The effect of immune factors, tumor necrosis factor- α, and agonistic autoantibodies to the angiotensin II type I receptor on soluble fms-like tyrosine-1 and soluble endoglin production in response to hypertension during pregnancy. Am J Hypertens 23: 911–916, 2010. doi:10.1038/AJH.2010.70. 20431529 PMC3500852

[179] Mydel P, Shipley JM, Adair-Kirk TL, Kelley DG, Broekelmann TJ, Mecham RP, Senior RM. Neutrophil elastase cleaves laminin-332 (laminin-5) generating peptides that are chemotactic for neutrophils. J Biol Chem 283: 9513–9522, 2008. doi:10.1074/JBC.M706239200. 18178964 PMC2442305

[180] Connelly JC, Skidgel RA, Schulz WW, Johnson AR, Erdös EG. Neutral endopeptidase 24.11 in human neutrophils: cleavage of chemotactic peptide. Proc Natl Acad Sci USA 82: 8737–8741, 1985. doi:10.1073/PNAS.82.24.8737. 3909153 PMC391512

[181] Walsh SW, Strauss JF. Pregnancy-specific expression of protease-activated receptor 1: a therapeutic target for prevention and treatment of preeclampsia? Am J Obstet Gynecol 226: S945–S953, 2022. doi:10.1016/J.AJOG.2021.11.1367. 35177224 PMC8868505

[182] Leik CE, Walsh SW. Neutrophils infiltrate resistance-sized vessels of subcutaneous fat in women with preeclampsia. Hypertension 44: 72–77, 2004. doi:10.1161/01.HYP.0000130483.83154.37. 15148293

[183] Krysiak O, Bretschneider A, Zhong E, Webb J, Hopp H, Verlohren S, Fuhr N, Lanowska M, Nonnenmacher A, Vetter R, Jankowski J, Paul M, Schönfelder G. Soluble vascular endothelial growth factor receptor-1 (sFLT-1) mediates downregulation of FLT-1 and prevents activated neutrophils from women with preeclampsia from additional migration by VEGF. Circ Res 97: 1253–1261, 2005. doi:10.1161/01.RES.0000194324.29363.82. 16269656

[184] Gordijn SJ, Beune IM, Thilaganathan B, Papageorghiou A, Baschat AA, Baker PN, Silver RM, Wynia K, Ganzevoort W. Consensus definition of fetal growth restriction: a Delphi procedure. Ultrasound Obstet Gynecol 48: 333–339, 2016. doi:10.1002/UOG.15884. 26909664

[185] Rosenfeld CS. Sex-specific placental responses in fetal development. Endocrinology 156: 3422–3434, 2015. doi:10.1210/EN.2015-1227. 26241064 PMC4588817

[186] Sundrani DP, Roy SS, Jadhav AT, Joshi SR. Sex-specific differences and developmental programming for diseases in later life. Reprod Fertil Dev 29: 2085–2099, 2017. doi:10.1071/RD16265. 28380326

[187] Tong W, Ganguly E, Villalobos-Labra R, Quon A, Spaans F, Giussani DA, Davidge ST. Sex-specific differences in the placental unfolded protein response in a rodent model of gestational hypoxia. Reprod Sci 30: 1994–1997, 2023. doi:10.1007/S43032-022-01157-W. 36574145 PMC10229681

[188] Tangri S, Wegmann TG, Lin H, Raghupathy R. Maternal anti-placental reactivity in natural, immunologically-mediated fetal resorptions. J Immunol 152: 4903–4911, 1994. doi:10.4049/JIMMUNOL.152.10.4903. 8176211

[189] Desforges M, Sibley CP. Placental nutrient supply and fetal growth. Int J Dev Biol 54: 377–390, 2010. doi:10.1387/IJDB.082765MD. 19876836

[190] Padmanabhan R, Singh S. Effect of natural litter size on the post-natal growth in CF rats. Acta Anat (Basel) 108: 436–442, 1980. doi:10.1159/000145342. 7270030

